# Strain, stress and rotation fields, and energetic features of twisted 2D materials

**DOI:** 10.1093/nsr/nwaf577

**Published:** 2025-12-16

**Authors:** Shuchang Li, Qian Zhang, Hanzheng Xing, Yuede Cao, Songyan Zhang, Xuan Zhang, Bin Ding, Xiaoyan Li

**Affiliations:** Mechano-X Institute, Applied Mechanics Laboratory, Department of Engineering Mechanics, Tsinghua University, Beijing 100084, China; Mechano-X Institute, Applied Mechanics Laboratory, Department of Engineering Mechanics, Tsinghua University, Beijing 100084, China; Mechano-X Institute, Applied Mechanics Laboratory, Department of Engineering Mechanics, Tsinghua University, Beijing 100084, China; Mechano-X Institute, Applied Mechanics Laboratory, Department of Engineering Mechanics, Tsinghua University, Beijing 100084, China; Mechano-X Institute, Applied Mechanics Laboratory, Department of Engineering Mechanics, Tsinghua University, Beijing 100084, China; Mechano-X Institute, Applied Mechanics Laboratory, Department of Engineering Mechanics, Tsinghua University, Beijing 100084, China; National Key Laboratory of Strength and Structural Integrity, Institute of Solid Mechanics, Beihang University, Beijing 100191, China; Mechano-X Institute, Applied Mechanics Laboratory, Department of Engineering Mechanics, Tsinghua University, Beijing 100084, China

**Keywords:** twisted 2D materials, nanoscale deformation, energetics, triangular partial-dislocation networks, hexagonal domains

## Abstract

Twisted two-dimensional (2D) materials exhibit remarkable quantum properties due to Moiré-pattern-induced electronic band structure change, highly sensitive to nanoscale deformation from atomic-scale reconstruction. The absence of an analytical model linking deformation to twist angle limits property tunability. We developed a theoretical model characterizing deformation and energetics of twisted 2D materials. As the twist angle increases, Moiré patterns evolve from triangular partial-dislocation networks to hexagonal domains with domain walls. Using anisotropic dislocation theory, we derived analytical expressions for local rotation, strain and stress fields at small twist angles, and a non-linear formula relating energy density to twist angle, capturing the transition from rapid growth to near saturation (0°–30°). Theoretical predictions agree well with previous experimental and computational studies and our atomistic simulations for twisted bilayer graphene, hexagonal boron nitride and trilayer graphene. This work provides a theoretical foundation for twist-angle control of quantum properties, enabling design of 2D quantum devices.

## Teaser

Based on anisotropic dislocation theory, a theoretical model was established to characterize the nanoscale deformation and energetic features of twisted two-dimensional materials, providing a theoretical basis for tuning their quantum properties.

## INTRODUCTION

Two-dimensional (2D) van der Waals (vdW) materials are composed of multiple strongly bonded atomic layers that are held together by relatively weak interlayer vdW forces [1]. As prominent examples of 2D vdW materials, few-layer graphene and hexagonal boron nitride (hBN) exhibit fascinating electrical, optical, thermal and mechanical properties [2–9]. When the neighboring layers of 2D materials are twisted relative to each other, a new type of 2D material (known as twisted 2D materials) emerges. The discovery of twisted 2D materials has opened a new era in condensed matter physics [10]. Numerous experimental studies have demonstrated that these materials exhibit unique Moiré superlattices and highly tunable physical properties [11–16]. These properties can be adjusted without altering their chemical composition by means of approaches such as electric field or substrate engineering [17,18]. For example, twisted bilayer graphene (tB-G) has highly tunable electronic properties that are sensitive to the twist angle [2–5]. At small twist angles, atomic-scale reconstruction occurs, resulting in changes in the interlayer commensurability and intralayer lattice distortion [19]. Such atomic-scale reconstruction significantly influences interlayer coupling and thereby alters the electronic band structure, resulting in intriguing transport properties and correlated electronic phases [3]. At the first ‘magic’ angle of ∼1.1°, unconventional superconductivity has been discovered, with the appearance of a flat band near zero Fermi energy [2]. Moreover, twisted 2D materials also exhibit other exceptional properties, including electromechanical effects [11–13], ferroelectricity [16], tunable optical properties [6], superlubricity [20–22], ultrahigh ballistic resistance [9] and anisotropic thermal transport [7,8].

These emergent properties of twisted 2D materials are closely linked to the periodicity of the Moiré superlattice and the structural deformation induced by atomic-scale reconstruction. Therefore, characterizing the deformation fields of the Moiré superlattice is paramount for understanding and controlling the emergent properties of twisted 2D materials. Bragg interferometry based on 4D scanning transmission electron microscopy (4D-STEM) has been used to capture atomic displacement fields in tB-G with twist angles smaller than 2° and to further calculate corresponding strain fields [14]. This is the first study to directly visualize the nanoscale deformations induced by atomic-scale reconstruction in twisted 2D materials. Recently, some theoretical studies [23–28] have also been performed to investigate the deformation and energetic features in twisted 2D materials. A continuum framework based on the classical linear elastic thin plate theory and generalized Peierls–Nabarro model was developed to describe the structural deformation of tB-G [26,27]. The non-linear Föppl‒von Kármán plate theory and density functional theory (DFT) calculations have been further incorporated into this framework to obtain more accurate results of out-of-plane deformation [28]. Furthermore, the Frenkel‒Kontorova model was developed to determine the structural behaviors in tB‒G [23,24,29]. Notably, these theoretical studies have focused mainly on the deformation fields in tB-G. Due to the intricate nature of the Moiré pattern, all these theoretical studies have provided numerical results through continuum modeling and atomistic simulations, with only a few studies offering semi-analytical expressions for displacement fields. To date, there have been no general full analytical solutions to deformation fields (including rotation, strain and stress fields) after atomic-scale reconstruction in twisted 2D materials, thereby impeding progress in the realm of twisted 2D materials.

In recent years, a number of experimental and computational studies [19,23–28,30–33] have shown that the structural deformation of twisted 2D materials is dominated by a dislocation network in the Moiré pattern. At small twist angles, the Moiré pattern consists of periodic commensurate domains separated by triangular incommensurate regions, which form straight lines accommodating interlayer lattice misfit at the vdW interfaces. These lines, known as vdW dislocations (or strain solitons) [31], define the dislocation network. In this study, we developed a theoretical model to describe the structural deformation and energetics of twisted 2D materials, exemplified by tB-G, twisted bilayer hBN (tB-hBN) and twisted trilayer graphene (tT-G), based on anisotropic dislocation theory. We first performed atomistic simulations to characterize their structural features. The results indicate that at small twist angles, the Moiré pattern is dominated by a triangular network of partial dislocations. Based on these results, we derived full analytical expressions for the strain, stress and rotation fields, which show good agreement with previous 4D-STEM experimental measurements on tB-G [14]. We also presented a non-linear formula linking energy density to twist angle, capturing the transition from rapid increase to near saturation in the range of 0° to 30°. The model’s predictions agree well with previous theoretical and computational studies [19,29,34,35] and with our atomistic simulations. This analytical model provides a fundamental insight into atomic-scale reconstruction and offers guidance for manipulating material properties through twist angle control.

## Atomistic simulations

**Figure 1.**
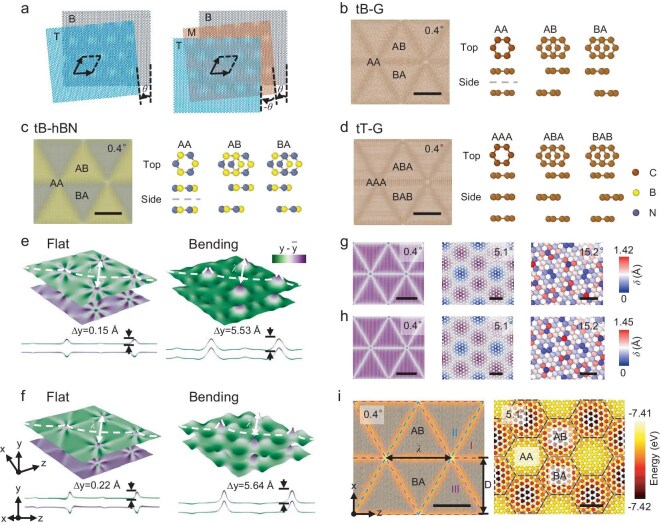
Atomic structures and energy maps of twisted systems from atomistic simulations. (a) Schematic diagram of tB-G and tT-G with *θ* = 5.1° before relaxation. (b–d) Triangular Moiré patterns in tB-G, tB-hBN and tT-G with *θ* = 0.4° after relaxation. The primary stacking sequences in tB-G and tB-hBN (AA, AB and BA stacking) and tT-G (AAA, ABA and BAB stacking) are shown in the top and side views. All scale bars are 20 nm. (e and f) Out-of-plane displacement of the flat and bending modes for tB-G and tB-hBN with *θ* = 0.4°, respectively. (g and h) Lattice mismatch in tB-G and tB-hBN with varying twist angles, quantified by using *δ*, respectively. The scale bars in tB-G and tB-hBN with *θ* = 0.4°, 5.1° and 15.2° are 20, 1 and 0.5 nm, respectively. (i) Energy maps of the top layer of tB-G with *θ* = 0.4° and 5.1°, respectively. The dislocations of Groups I, II and III are represented by red, blue and purple dashed lines, respectively, in tB-G with* θ* = 0.4°. The domain walls are indicated by black dashed lines in tB-G with* θ* = 5.1°. The scale bars in tB-G with *θ* = 0.4° and 5.1° are 20 and 1 nm, respectively.

Before developing a theoretical model of twisted 2D materials, we performed a series of atomistic simulations to investigate the structural deformation of typical twisted 2D materials (including tB-G, tB-hBN and tT-G). Figure 1a shows the atomic structures of tB-G and tT-G with a twist angle of *θ* = 5.1° before relaxation, which were used for atomistic simulation. These simulated samples have hexagonal Moiré patterns with periodicities of *λ*, which depends on *θ*  as *λ* = *a*/[2 sin(*θ*/2)], where *a* is the lattice constant. Figure 1b–d shows the triangular Moiré patterns in tB-G, tB-hBN and tT-G with *θ* = 0.4° after relaxation, and their corresponding top and side views of different stackings. At small twist angles, the simulated samples experience significant atomic-scale reconstruction during relaxation, which reduces the interlayer misfit energy but at the cost of intralayer elastic energy. The reconstruction process involves the expansion of energetically favorable AB/BA stacking regions and the shrinkage of energetically unfavorable AA stacking regions, as well as out-of-plane deformation, eventually resulting in the formation of triangular Moiré patterns [3] (Fig. 1b and c). Figure 1e and f shows the out-of-plane displacement contours of the flat and bending modes for relaxed tB-G and tB-hBN with *θ* = 0.4° and their corresponding slice views along the dashed lines, respectively. In flat mode, the top and bottom layers buckle out of plane in opposite directions at the AA-nodes. However, in the bending mode, both layers bulge along the same directions at the AA-nodes. For both tB-G and tB-hBN, the maximum deformation amplitude of the bending mode is approximately 25–35 times greater than that of the flat mode. The bending mode has a slightly lower energy than the flat mode at small twist angles [19,35], but involves a more intricate deformation. Given that the flat mode can occur at both small and large twist angles, we focus primarily on the flat mode in the subsequent theoretical model. We also examined the flat mode of tT-G with *θ* = 0.4° (Fig. S1). Similar to the behaviors in tB-G, the top and bottom layers exhibit out-of-plane buckling in opposite directions at the AA-nodes with small deformation amplitude, while the middle layer remains largely planar with negligible out-of-plane displacement.

Figure 1g and h presents the lattice mismatch contours in tB-G and tB-hBN with *θ* = 0.4°, 5.1° and 15.2°, rendered via lateral distance *δ*  between the nearest atoms in two adjacent layers, respectively. At small twist angles, the Moiré pattern consists of a periodic array of AA-stacking regions connected by partial screw dislocation lines that enclose triangular regions with AB/BA stacking. In AA stacking regions, atoms in the top layer directly overlap with atoms in the bottom layer, i.e. *δ* = 0. In AB/BA regions, atoms of two layers are overlapped or mismatched by a bond length, i.e. *δ* = 0 or *δ* = *a*/√3. In partial dislocation lines, atoms of two layers are mismatched by a half-bond length, i.e. *δ* = *a*/2√3, resulting in Burgers vectors with a magnitude of *b*_s_ = *a*/2√3. At large twist angles, the Moiré pattern consists of periodic hexagonal domains separated by domain walls, which is related to insignificant atomic-scale reconstruction. As the twist angle increases, the Moiré pattern gradually transitions from a triangular dislocation network to a hexagonal network. Figure 1i shows the atomic potential energy maps in tB-G with *θ* = 0.4° and 5.1°. For small twist angles, the dislocation lines have relatively high potential energy. According to different line vectors, we categorized the dislocations into three groups (Groups I, II and III), which are indicated by the red, blue and purple dashed lines in Fig. 1i, respectively. The Burgers vectors of the three groups of dislocation lines have different directions but the same magnitude. At large twist angles, AA-domains and domain walls have higher potential energy regions. These results suggest that the energy change of the simulated sample with a small *θ* is mainly due to the formation of a triangular dislocation network, whereas at larger *θ*, it is primarily associated with AA-domains and domain walls.

## Theoretical model

### Analytical expressions for rotation, strain and stress fields in twisted 2D materials after local reconstruction

Based on the structural deformation and relevant features of twisted 2D materials from atomistic simulations, we derived analytical expressions of local reconstruction displacement, rotation, strain and stress fields in twisted 2D materials with a small *θ* via the anisotropic dislocation theory. Notably, the results from atomistic simulations are mainly used to compare with the predictions from our subsequent theoretical model. Atomistic simulations indicated the presence of a triangular dislocation network of twisted 2D materials with the small twist angle, which gives us a hint for establishing the theoretical model. However, our theoretical model is based on the anisotropic dislocation theory in the framework of continuum mechanics, and does not require specific atomic coordinates from atomistic simulations. Owing to their weak interlayer vdW interactions and robust intralayer covalent-bonded interactions, 2D materials are generally regarded as transversely isotropic materials. As shown in Fig. 1i, partial screw dislocations in twisted bilayer systems are categorized into three groups, labeled Groups I, II and III, with each group oriented 60° apart. Note that each group consists of an infinite number of parallel partial dislocations with screw features, and the displacement fields of the entire triangular network of dislocations can be obtained by summing the displacement fields of each individual screw dislocation within three groups.

Thus, three components in Cartesian coordinates (*x, y, z*) of the displacement field are expressed as:


(1) \begin{eqnarray*}
{u}_x &=& - \displaystyle\frac{{\sqrt 3 {b}_s}}{{4\pi }}\sum\limits_{n = - \infty }^\infty {\arctan\! \left( {\displaystyle\frac{{\eta y}}{{\left( {\frac{1}{2}x - \frac{{\sqrt 3 }}{2}z} \right) + nD}}} \right)} \nonumber\\
&&-\, \arctan\! \left( {\frac{{\eta y}}{{\left( {\frac{1}{2}x + \frac{{\sqrt 3 }}{2}z} \right) + nD}}} \right), \nonumber\\
{u}_y &=& 0,\nonumber\\
{u}_z &=& -\, \displaystyle\frac{{{b}_s}}{{2\pi }}\sum\limits_{n = - \infty }^\infty \arctan\! \left( {\displaystyle\frac{{\eta y}}{{x + nD}}} \right) \nonumber\\
&&+\, \frac{1}{2}\arctan\! \left( {\frac{{\eta y}}{{\left( {\frac{1}{2}x - \frac{{\sqrt 3 }}{2}z} \right) + nD}}} \right) \nonumber\\
&&+\, \frac{1}{2}\arctan\! \left( {\frac{{\eta y}}{{\left( {\frac{1}{2}x + \frac{{\sqrt 3 }}{2}z} \right) + nD}}} \right), \end{eqnarray*}

where *b_s_* is the magnitude of the Burgers vector of partial dislocation (*b*_s_ = *a*/√3 in the current system) and *D* is the distance between adjacent parallel dislocation lines (*D* = √3*λ*/2). In Equation (1), *η* is an anisotropy parameter and is given by:


(2) \begin{equation*}{\eta }^2 = \frac{1}{2}\left( {{C}_{11} - {C}_{12}} \right)/{C}_{44},\end{equation*}

where *C*_11_, *C*_12_ and *C*_44_ represent three independent elastic constants of bilayer 2D materials with hexagonal symmetry. Here we emphasize the coordinate system (*x, y, z*) used for displacement, and subsequent rotation, strain and stress fields in twisted bilayer systems. The *xz*-plane is located at the midplane between top and bottom layers in the twisted bilayer system and aligns with the dislocation network. The *y*-axis is set orthogonal to the atomic layers. The origin is established at a junction point of three groups of dislocation lines in a single periodic triangular dislocation network, corresponding to the projection of an AA-node’s center onto the midplane.

According to the geometric equation and the summation of infinite series [36], we obtained the following expression of the total in-plane rotation angle *ϕ*_T_:


(3) \begin{eqnarray*}
{\phi }_{\mathrm{T}} &=& \displaystyle\frac{1}{2}\left( {\displaystyle\frac{{\partial {u}_z}}{{\partial x}} - \displaystyle\frac{{\partial {u}_x}}{{\partial z}}} \right) \nonumber\\
&=& \displaystyle\frac{{{b}_s}}{{4D}}\left\{ \begin{array}{@{}l@{}}
\displaystyle\frac{{\sinh \left( {2\pi \frac{{\eta y}}{D}} \right)}}{{\cosh \left( {2\pi \frac{{\eta y}}{D}} \right) - \cos \left( {2\pi \frac{x}{D}} \right)}} \\
+\, \displaystyle\frac{{\sinh \left( {2\pi \frac{{\eta y}}{D}} \right)}}{{\cosh \left( {2\pi \frac{{\eta y}}{D}} \right) - \cos \left( {\pi \frac{{x - \sqrt 3 z}}{D}} \right)}}\\
+\, \displaystyle\frac{{\sinh \left( {2\pi \frac{{\eta y}}{D}} \right)}}{{\cosh \left( {2\pi \frac{{\eta y}}{D}} \right) - \cos \left( {\pi \frac{{x + \sqrt 3 z}}{D}} \right)}} \end{array} \right\}.\nonumber\\
\end{eqnarray*}

By eliminating the initial twist angle *θ*, we further obtained the following expression of local rotation angle *ϕ*_R_ of the top layer relative to the bottom layer in twisted bilayer systems:


(4) \begin{equation*}{\phi }_{\mathrm{R}}\!\left( {x,z} \right) = {\phi }_{\mathrm{T}}\!\left( {x,\frac{h}{2},z} \right) - {\phi }_{\mathrm{T}}\!\left( {x, - \frac{h}{2},z} \right) - \theta. \end{equation*}

Using the geometric equation and the summation of infinite series [36], we further obtained the following strain field with six components of the twisted bilayer system:


(5) \begin{eqnarray*}
{\varepsilon }_{xx} &=& \frac{{\sqrt 3 {b}_s}}{{8D}}\left\{ {\frac{{\sinh \!\left( {2\pi \frac{{\eta y}}{D}} \right)}}{{\cosh \!\left( {2\pi \frac{{\eta y}}{D}} \right) - \cos \!\left( {\pi \frac{{x - \sqrt 3 z}}{D}} \right)}}} \right.\nonumber \\
&&\left. -\, {\frac{{\sinh \!\left( {2\pi \frac{{\eta y}}{D}} \right)}}{{\cosh \!\left( {2\pi \frac{{\eta y}}{D}} \right) - \cos \!\left( {\pi \frac{{x + \sqrt 3 z}}{D}} \right)}}} \right\},\nonumber\\
{\varepsilon }_{yy} &=& 0, \nonumber\\
{\varepsilon }_{zz} &=& - \frac{{\sqrt 3 {b}_s}}{{8D}}\left\{ {\frac{{\sinh \!\left( {2\pi \frac{{\eta y}}{D}} \right)}}{{\cosh \!\left( {2\pi \frac{{\eta y}}{D}} \right) - \cos \!\left( {\pi \frac{{x - \sqrt 3 z}}{D}} \right)}} }\right.\nonumber\\
&&\left. {-\, \frac{{\sinh \!\left( {2\pi \frac{{\eta y}}{D}} \right)}}{{\cosh \!\left( {2\pi \frac{{\eta y}}{D}} \right) - \cos \!\left( {\pi \frac{{x + \sqrt 3 z}}{D}} \right)}}} \right\},\nonumber\\
{\varepsilon }_{yz} &=& - \frac{{{b}_s}}{{4D}} \cdot \eta \left\{ {\frac{{\sin \!\left( {2\pi \frac{x}{D}} \right)}}{{\cosh \!\left( {2\pi \frac{{\eta y}}{D}} \right) - \cos \!\left( {2\pi \frac{x}{D}} \right)}}}\right. \nonumber\\
&& +\, \frac{1}{2}\frac{{\sin \!\left( {\pi \frac{{x - \sqrt 3 z}}{D}} \right)}}{{\cosh \!\left( {2\pi \frac{{\eta y}}{D}} \right) - \cos \!\left( {\pi \frac{{x - \sqrt 3 z}}{D}} \right)}} \nonumber\\
&&\left.{+\, \frac{1}{2}\frac{{\sin \!\left( {\pi \frac{{x + \sqrt 3 z}}{D}} \right)}}{{\cosh \!\left( {2\pi \frac{{\eta y}}{D}} \right) - \cos \!\left( {\pi \frac{{x + \sqrt 3 z}}{D}} \right)}}} \right\},\nonumber\\
{\varepsilon }_{xz} &=& \frac{{{b}_s}}{{4D}}\left\{ {\frac{{\sinh \left( {2\pi \frac{{\eta y}}{D}} \right)}}{{\cosh \left( {2\pi \frac{{\eta y}}{D}} \right) - \cos \left( {2\pi \frac{x}{D}} \right)}}}\right. \nonumber\\
&&-\, \frac{1}{2}\frac{{\sinh \left( {2\pi \frac{{\eta y}}{D}} \right)}}{{\cosh \left( {2\pi \frac{{\eta y}}{D}} \right) - \cos \left( {\pi \frac{{x - \sqrt 3 z}}{D}} \right)}} \nonumber\\
&&\left. {-\, \frac{1}{2}\frac{{\sinh \left( {2\pi \frac{{\eta y}}{D}} \right)}}{{\cosh \left( {2\pi \frac{{\eta y}}{D}} \right) - \cos \left( {\pi \frac{{x + \sqrt 3 z}}{D}} \right)}}} \right\},\nonumber\\
{\varepsilon }_{xy} &=& - \frac{{\sqrt 3 {b}_s}}{{8D}} \cdot \eta \left\{ {\frac{{\sin \left( {\pi \frac{{x - \sqrt 3 z}}{D}} \right)}}{{\cosh \left( {2\pi \frac{{\eta y}}{D}} \right) - \cos \left( {\pi \frac{{x - \sqrt 3 z}}{D}} \right)}}}\right. \nonumber\\
&&\left. {-\, \frac{{\sin \left( {\pi \frac{{x + \sqrt 3 z}}{D}} \right)}}{{\cosh \left( {2\pi \frac{{\eta y}}{D}} \right) - \cos \left( {\pi \frac{{x + \sqrt 3 z}}{D}} \right)}}} \right\}. \end{eqnarray*}

By substituting Equation (5) into the expression of the maximum in-plane shear strain ${\gamma }_{\max } = 2\sqrt {{{{{( {{\varepsilon }_{xx} - {\varepsilon }_{zz}} )}}^2} \mathord{/ {\vphantom {{{{( {{\varepsilon }_{xx} - {\varepsilon }_{zz}} )}}^2} 4}} } 4} + {{( {{\varepsilon }_{xz}} )}}^2} $, we can calculate the maximum in-plane shear strain of the twisted bilayer system.

Both the bilayer graphene and hBN systems have five independent elastic constants (*C*_11_, *C*_12_, *C*_13_, *C*_33_ and *C*_44_). These elastic constants were determined by fitting the stress‒strain curves under small strain from atomistic simulations and are listed in Table S1. Based on the generalized Hooke’s law, we obtained the following stress field with six components:


(6) \begin{eqnarray*}
{\sigma }_{xx} &=& \frac{{\sqrt 3 {b}_s}}{{4D}} \cdot {K}_s\eta \left\{ {\frac{{\sinh \left( {2\pi \frac{{\eta y}}{D}} \right)}}{{\cosh \left( {2\pi \frac{{\eta y}}{D}} \right) - \cos \left( {\pi \frac{{x - \sqrt 3 z}}{D}} \right)}} }\right. \nonumber\\
&& \left.{-\, \frac{{\sinh \left( {2\pi \frac{{\eta y}}{D}} \right)}}{{\cosh \left( {2\pi \frac{{\eta y}}{D}} \right) - \cos \left( {\pi \frac{{x + \sqrt 3 z}}{D}} \right)}}} \right\},\nonumber\\
{\sigma }_{yy} &=& 0, \nonumber\\
{\sigma }_{zz} &=& - \frac{{\sqrt 3 {b}_s}}{{4D}} \cdot {K}_s\eta \left\{ {\frac{{\sinh \left( {2\pi \frac{{\eta y}}{D}} \right)}}{{\cosh \left( {2\pi \frac{{\eta y}}{D}} \right) - \cos \left( {\pi \frac{{x - \sqrt 3 z}}{D}} \right)}}}\right. \nonumber\\
&&\left.{ -\, \frac{{\sinh \left( {2\pi \frac{{\eta y}}{D}} \right)}}{{\cosh \left( {2\pi \frac{{\eta y}}{D}} \right) - \cos \left( {\pi \frac{{x + \sqrt 3 z}}{D}} \right)}}} \right\},\nonumber\\
{\sigma }_{yz} &=& - \frac{{{b}_s}}{{2D}} \cdot {K}_s\left\{ {\frac{{\sin \left( {2\pi \frac{x}{D}} \right)}}{{\cosh \left( {2\pi \frac{{\eta y}}{D}} \right) - \cos \left( {2\pi \frac{x}{D}} \right)}}}\right.\nonumber\\
&& +\, \frac{1}{2}\frac{{\sin \left( {\pi \frac{{x - \sqrt 3 z}}{D}} \right)}}{{\cosh \left( {2\pi \frac{{\eta y}}{D}} \right) - \cos \left( {\pi \frac{{x - \sqrt 3 z}}{D}} \right)}}\nonumber\\
&&\left. {+\, \frac{1}{2}\frac{{\sin \left( {\pi \frac{{x + \sqrt 3 z}}{D}} \right)}}{{\cosh \left( {2\pi \frac{{\eta y}}{D}} \right) - \cos \left( {\pi \frac{{x + \sqrt 3 z}}{D}} \right)}}} \right\},\nonumber\\
{\sigma }_{xz} &=& \frac{{{b}_s}}{{2D}} \cdot {K}_s\eta \left\{ {\frac{{\sinh \left( {2\pi \frac{{\eta y}}{D}} \right)}}{{\cosh \left( {2\pi \frac{{\eta y}}{D}} \right) - \cos \left( {2\pi \frac{x}{D}} \right)}}}\right. \nonumber\\
&&-\, \frac{1}{2}\frac{{\sinh \left( {2\pi \frac{{\eta y}}{D}} \right)}}{{\cosh \left( {2\pi \frac{{\eta y}}{D}} \right) - \cos \left( {\pi \frac{{x - \sqrt 3 z}}{D}} \right)}}\nonumber\\
&&\left. {-\, \frac{1}{2}\frac{{\sinh \left( {2\pi \frac{{\eta y}}{D}} \right)}}{{\cosh \left( {2\pi \frac{{\eta y}}{D}} \right) - \cos \left( {\pi \frac{{x + \sqrt 3 z}}{D}} \right)}}} \right\},\nonumber\\
{\sigma }_{xy} &=& - \frac{{\sqrt 3 {b}_s}}{{4D}} \cdot {K}_s\left\{ {\frac{{\sin \left( {\pi \frac{{x - \sqrt 3 z}}{D}} \right)}}{{\cosh \left( {2\pi \frac{{\eta y}}{D}} \right) - \cos \left( {\pi \frac{{x - \sqrt 3 z}}{D}} \right)}}}\right.\nonumber\\
&&\left. { -\, \frac{{\sin \left( {\pi \frac{{x + \sqrt 3 z}}{D}} \right)}}{{\cosh \left( {2\pi \frac{{\eta y}}{D}} \right) - \cos \left( {\pi \frac{{x + \sqrt 3 z}}{D}} \right)}}} \right\},
\end{eqnarray*}

where *K_s_* is called an energy coefficient and expressed as follows:


(7) \begin{equation*}{K}_s = {\left[ {\frac{1}{2}\left( {{C}_{11} - {C}_{12}} \right){C}_{44}} \right]}^{\frac{1}{2}}.\end{equation*}

By taking the partial derivative of Equation (5), we derived the strain gradient field, as detailed in Equation (S21). The complete derivations of the analytical expressions for the local reconstruction rotation, strain, stress, and strain gradient fields in twisted bilayer systems are provided in Section S1.1. We also derived the rotation, strain and stress fields of twisted trilayer systems, which are shown in Section S1.3. Notably, the current theoretical model for strain, stress and rotation fields is only applicable to twisted few-layer systems with small twist angles. At large twist angles, atomic-scale reconstruction is weakened, while the increased prominence of AA-nodes becomes a crucial factor that must be considered. Consequently, the Moiré superlattice at large twist angles cannot be adequately characterized by using the current model based on three sets of screw dislocations.

### Analytical expressions of energy density in twisted 2D materials

The energy landscapes (Fig. 1i) of tB-G with *θ* = 0.4° and *θ* = 5.1° indicate that both the AA-nodes and the dislocation lines exhibit elevated potential energy. For a small twist angle (*θ* < *θ_c_*, where *θ_c_* is a critical twist angle), the AA-nodes are relatively small, and their influence on the system energy can be negligible. Therefore, the energy of the system with a small twist angle is dominated by a triangular dislocation network. Figure 1g and h shows that as the twist angle increases, the relative size of the AA-node gradually increases. For the large twist angle (*θ* > *θ_c_*), the AA-nodes expand into the AA-domains, with the formation of hexagonal domain walls among these AA-domains (Fig. 1g and h). The AA-domains are identified as the circular regions where the value of lattice mismatch *δ  *of each atom is less than half the magnitude of the dislocation Burgers vector (i.e. *δ* < *b*_s_/2). Thus, the energy of the system with a large twist angle is determined by the AA-domains and their corresponding domain walls.

For a small twist angle (*θ* < *θ_c_*), the energy of the twisted bilayer system is equal to the sum of the interaction energy between two dislocations in the triangular dislocation network and the self-energy of the dislocation line. By evaluating the interaction energy between two dislocations through integration and adding the dislocation core energy, we obtained the following energy density (*γ*_twist_) of the entire system:


(8) \begin{eqnarray*}
{\gamma }_{{\mathrm{twist}}} &=& \frac{{{K}_{\mathrm{s}}a}}{{2\sqrt 3 \pi }} \nonumber\\
&&\cdot\, \theta\! \left\{ {\frac{{2\pi \eta {r}_0}}{{\sqrt 3 a}}\theta - \ln \left[ {\sinh \left( {\frac{{2\pi \eta {r}_0}}{{\sqrt 3 a}}\theta } \right)} \right] - \ln 2}\! \right\} \nonumber\\
&&+ \frac{{2\sqrt 3 {E}_{\mathrm{c}}}}{a} \cdot \theta,\end{eqnarray*}

where *r*_0_ is the dislocation core radius and *E_c_* is the core energy per unit length. In Equation (8), the first term represents the interaction energy between two dislocations and is a non-linear function of *θ*, and the second term represents the dislocation core energy, which is linearly proportional to *θ*.

For a large twist angle (*θ* > *θ_c_*), the energy of the twisted bilayer system encompasses the energies of the AA-domains and domain walls. Assuming that the energy density *γ*_AA_ of the AA-domain and the energy per unit length *E*_dw_ of the domain wall are constant, we obtained the total energy density of the system as:


(9) \begin{equation*}{\gamma }_{{\mathrm{twist}}} = \frac{{4\sqrt 3 {E}_{{\mathrm{dw}}}}}{a} \cdot \sin \left( {\frac{\theta }{2}} \right) + \frac{1}{3}{\gamma }_{{\mathrm{AA}}}.\end{equation*}

The detailed derivations of Equations (8) and (9) are provided in Section S1.2. Combining Equations (8) and (9), we wrote the total energy density of the twisted bilayer system with hexagonal symmetry as:


(10) \begin{eqnarray*}
&&{\gamma }_{{\mathrm{twist}}} = \nonumber\\
&&\left\{ \begin{array}{@{}l@{}} \frac{{{K}_{\mathrm{s}}a}}{{2\sqrt 3 \pi }} \cdot \theta \left\{ {\frac{{2\pi \eta {r}_0}}{{\sqrt 3 a}}\theta - \ln \left[ {\sinh \left( {\frac{{2\pi \eta {r}_0}}{{\sqrt 3 a}}\theta } \right)} \right] - \ln 2} \right\}\nonumber\\
\quad +\, \frac{{2\sqrt 3 {E}_{\mathrm{c}}}}{a} \cdot \theta ,\quad \theta \in [0,{\theta }_{\mathrm{c}}{\mathrm{) }}\nonumber\\
\frac{{4\sqrt 3 {E}_{{\mathrm{dw}}}}}{a} \cdot \sin \left( {\frac{\theta }{2}} \right) + \frac{1}{3}{\gamma }_{{\mathrm{AA}}},\quad \theta \in [{\theta }_{\mathrm{c}},\ \frac{\pi }{6}].\ \end{array} \right.\nonumber\\
\end{eqnarray*}

The twisted trilayer system exhibits nearly the same energetic features as the twisted bilayer system, as shown in the Fig. S2c and S2d. Note that there are two dislocation networks in the twisted trilayer system, and both the total energy and interfacial area of the twisted trilayer system are double those of the twisted bilayer system.

## RESULTS

### Atomic-scale reconstruction of twisted 2D materials

**Figure 2.**
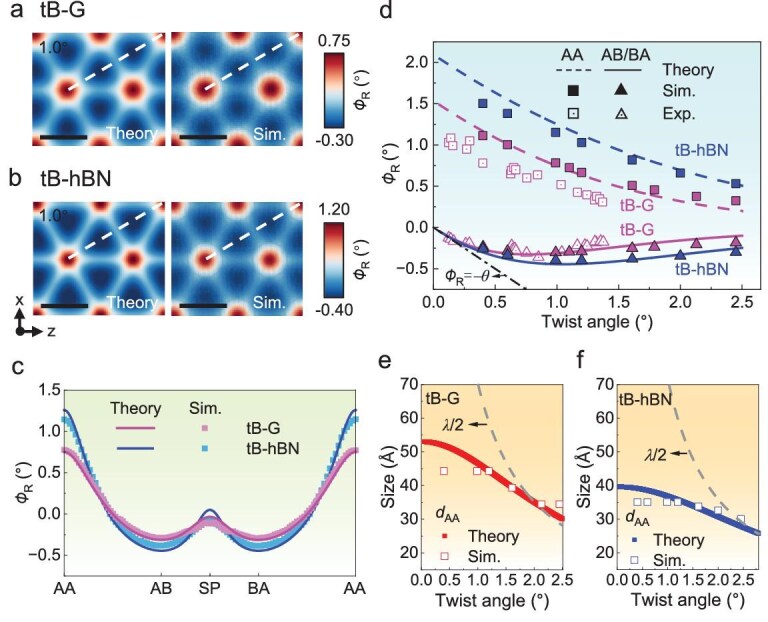
Local rotation fields in tB-G and tB-hBN. (a and b) Contours of *ϕ*_R_ in tB-G and tB-hBN with* θ* = 1.0° from the theoretical model and atomistic simulations (Sim.). All scale bars are 10 nm. (c) Variation in *ϕ*_R_ along the dashed lines shown in (a) and (b). (d) *ϕ*_R_ in the AA and AB/BA stacking regions as a function of the twist angle from the theoretical model, atomistic simulations and experiments (Exp.) [14]. The data for tB-G and tB-hBN are shown in pink and blue, respectively. The theoretical and simulation results for tB-G and tB-hBN correspond to *ϕ*_R_ at the centers of the AA and AB stacking regions. The experimental results for tB-G represent the average *ϕ*_R_ obtained from a 4D-STEM dataset over a homogeneous twist-angle region [14]. (e and f) Diameters of the AA-nodes as functions of the twist angle* θ* from the theoretical model and atomistic simulations. The dashed lines represent the variation in *λ*/2 with *θ* in tB-G and tB-hBN.

Figure 2a and b shows comparisons of the contours of *ϕ*_R_ in the top layers of tB-G and tB-hBN with *θ* = 1.0° obtained from our theoretical model and atomistic simulations, respectively. A positive *ϕ*_R_ indicates that the local rotation aligns with the initial rigid rotation, whereas a negative *ϕ*_R_ suggests that the local rotation occurs in the opposite direction. The twisted bilayer system has three primary stacking sequences: energetically favorable AB and BA stacking and unfavorable AA stacking. As shown in Fig. 2a and b, *ϕ*_R_ in the AB and BA stacking regions is negative, whereas *ϕ*_R_ in the AA stacking regions is positive. In tB-G and tB-hBN, the AB/BA stacking regions possessing negative *ϕ*_R_ grow more commensurate during reconstruction by counteracting the initial rigid rotation *θ*. In contrast, AA stacking regions featuring positive *ϕ*_R_ gradually shrink into localized AA-nodes during reconstruction. Figure 2c shows the variations in *ϕ*_R_ along the dashed lines shown in Fig. 2a and b obtained from our theoretical model and atomistic simulations. Our theoretical predictions show excellent agreement with atomistic simulations. The dashed lines in Fig. 2a and b sequentially traverse through the AA, AB, saddle point (SP) and BA stacking regions, spanning across an entire superlattice. The SP stacking corresponds to the dislocation line. In Fig. 2c, *ϕ*_R_ attains its maximum and minimum values at the centers of the AA and AB/BA stacking regions, respectively. Its local maxima in the SP stacking regions are approximately zero.

Figure 2d shows the values of *ϕ*_R_ at the centers of the AB/BA stacking regions and AA-nodes (denoted as *ϕ*_R-AB/BA_ and *ϕ*_R-AA_, respectively) in the top layers of tB-G and tB-hBN as functions of *θ* from our theoretical model and atomistic simulations. As *θ* increases, *ϕ*_R-AA_ monotonically decreases and tends toward zero at *θ* = 2.5°, beyond which the degree of atomic-scale reconstruction becomes almost completely diminished. However, *ϕ*_R-AB/BA_ initially decreases and then increases as *θ* increases, ultimately approaching zero at *θ* = 2.5°. Notably, the absolute value of *ϕ*_R-AB/BA_ approaches the value of *θ* at *θ* < 0.2°, implying that the initial twist near the centers of the AB/BA stacking regions is counterbalanced by significant rotational reconstruction, leading to nearly complete reversion to the commensurate configurations after full relaxation. Figure 2d shows that the predictions for both tB-G and tB-hBN from our theoretical model agree well with the results from atomistic simulations. However, the experimental results for tB-G exhibit lower absolute values than the theoretical and simulation results. The experimental results for tB-G are the average *ϕ*_R_ obtained from a 4D-STEM dataset over a homogeneous twist-angle region [14], which has lower absolute values than *ϕ*_R_ at the centers of the corresponding stacking regions. Notably, the theoretical, simulation and experimental results still maintain consistent variation trends with increasing *θ*. We further compared the local rotation fields *ϕ*_R_ in the bottom and middle layers of tT-G with* θ* = 0.4° from our theoretical model and atomistic simulations, as shown in Fig. S3. The predictions from our model are consistent with the results from our simulations. The significant differences in *ϕ*_R_ in Fig. S3c between our theoretical model and atomistic simulations arise from ignoring the interlayer interaction between the top and bottom layers in our model for the twisted trilayer system.

Following the topological definition of AA-nodes [34], we calculated the diameter *d*_AA_ of the AA-nodes as follows:


(11) \begin{equation*}
{d}_{{\mathrm{AA}}} = \frac{{{b}_s}}{{2\left| {\phi _{{\mathrm{T \hbox{-} AA}}}^{{\mathrm{tB \hbox{-} Top}}}} \right|}},\end{equation*}

where $\phi _{{\mathrm{T \hbox{-} AA}}}^{{\mathrm{tB \hbox{-} Top}}}$ represents the total rotation angle at the center of the AA-nodes of the top layer of the twisted bilayer system. Figure 2e and f shows the values of *d*_AA_ as functions of *θ* in tB-G and tB-hBN, respectively. As *θ* increases, both the superlattice period *λ* and the AA-node size *d*_AA_ decrease gradually. When *d*_AA_ reaches up to half of the superlattice period *λ/*2, the Moiré pattern of the twisted bilayer system mainly consists of AA-domains and their corresponding domain walls. This intersection occurs at ${\theta }_{\lambda = 2{d}_{{\mathrm{AA}}}} = 2.02^\circ $ and ${\theta }_{\lambda = 2{d}_{{\mathrm{AA}}}} = 2.76^\circ $ for tB-G and tB-hBN, respectively. As the size of the AA-nodes increases, their impact on energy becomes more significant and cannot be neglected. Therefore, the expression *d*_AA_ = *λ*/2 provides a reliable prediction for determining the critical twist angle *θ*_c_ that separates the small and large twist regimens. Notably, the theoretical predictions of *d*_AA_ align well with simulation results except at relatively small *θ*. The observed discrepancy in *d*_AA_ between theoretical model and atomistic simulations arises from the neglect of out-of-plane deformation [34]. As shown in Fig. S4, the out-of-plane displacements increase as the twist angle decreases below 1.0°, deviating from the flat-structure assumption of the model and leading to a more pronounced discrepancy in *d*_AA_ at smaller* θ*.

### Features of strain and stress fields in twisted 2D materials

**Figure 3.**
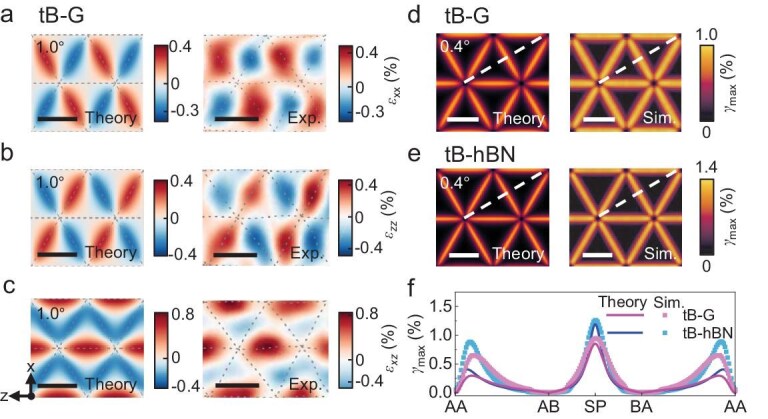
Strain fields in tB-G and tB-hBN. (a–c) Contours of in-plane strain components (*ε_xx_, ε_zz_* and *ε_xz_*) in the top layer of tB-G with *θ* = 1.0° from the theoretical model and experiments (experimental results are reproduced with permission from Kazmierczak *et al.* [14]). All scale bars are 10 nm. (d and e) Maximum in-plane shear strain contours in the top layer of tB-G and tB-hBN with *θ* = 0.4° from the theoretical model and atomistic simulations. All scale bars are 20 nm. (f) Variations in the maximum in-plane shear strain along the dashed lines shown in (d) and (e).

Figure 3a–c shows the contours of in-plane strain components (*ε_xx_, ε_zz_* and *ε_xz_*) in the top layer of tB-G with *θ* = 1.0° from our theoretical model and previous experiments [14]. Owing to the inevitable local tensile or compressive deformation during the experiment, the experimental results are partially distorted but are consistent with our theoretical prediction. The distribution of the in-plane strain components exhibits central symmetry about the center of the AA-nodes. Near the dislocation lines, atoms from adjacent AB and BA stacking regions move in opposite directions during reconstruction, inducing significant shear deformation. This deformation results in high strain levels, further contributing to the overall structural changes. The fields of *γ*_max_ in tB-G and tB-hBN with *θ* = 0.4°, as obtained from the theoretical model and atomistic simulations, are shown in Fig. 3d and e. The fields of *γ*_max_ exhibit 6-fold symmetry, with their maximum value occurring at the midpoint of adjacent AA-nodes. We extracted the data along the path depicted by the white dashed lines in Fig. 3d and e and plotted them in Fig. 3f. The maximum values occur at the dislocation lines (SP stacking), where the most significant shear deformation takes place, with values of approximately 1.0% and 1.4% for tB-G and tB-hBN, respectively, with *θ* = 0.4°. *γ*_max_ near the dislocation lines from atomistic simulations is nearly identical to that from the theoretical model. However, near the AA-nodes, atomistic simulations calculate *γ*_max_ to be about twice the theoretical value, primarily due to the larger interlayer spacing of AA stacking compared to AB/BA stacking. This results in significant out-of-plane deformation near AA-nodes being experienced during relaxation. Given the relatively small size of the AA-nodes, it is reasonable to ignore their influence for convenience. The AB/BA stacking regions, however, exhibit minimal deformation, with *γ*_max_ in these regions being nearly zero according to both the theoretical model and atomistic simulations. We further compared the distributions of *γ*_max_ of tB-G with *θ* = 1.1° between our theoretical model and previous experiments [14] in Fig. S5. Although the experimental results are distorted due to complicated deformation, the contour of *γ*_max_ predicted from our theoretical model is similar to that from previous experimental results. We also investigated the feature of strain fields in tT-G by using the theoretical model and atomistic simulations (Fig. S6). Notably, the theoretical results exhibit excellent agreement with the atomistic simulation results for the bilayer systems, tB-G and tB-hBN, as well as the twisted trilayer system tT-G.

**Figure 4.**
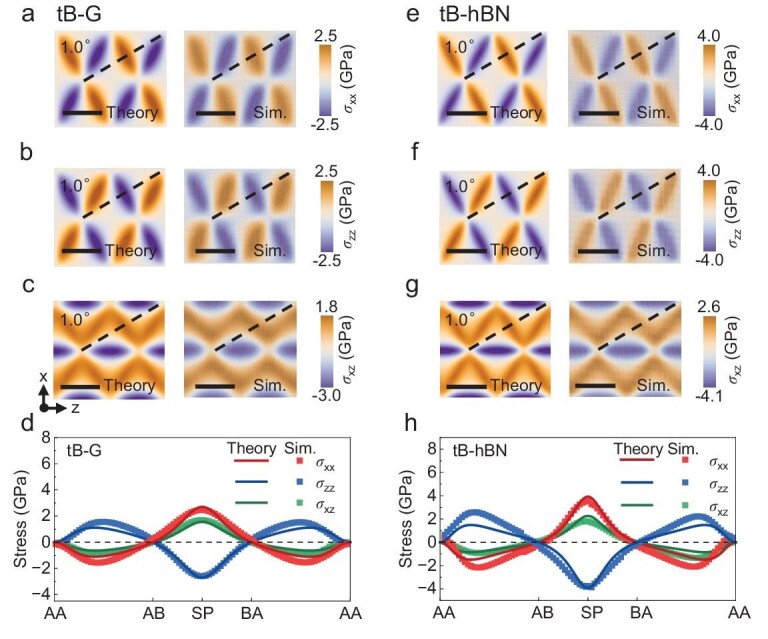
Stress components in tB-G and tB-hBN. (a–c) Contours of in-plane stress components (*σ_xx_, σ_zz_* and *σ_xz_*) in the top layer of tB-G with *θ* = 1.0° from the theoretical model and atomistic simulations. All scale bars are 10 nm. (d) Variations in the stress components (*σ_xx_, σ_zz_* and *σ_xz_*) at tB-G along the dashed lines shown in (a*–*c). (e–g) Contours of in-plane stress components (*σ_xx_, σ_zz_* and *σ_xz_*) in the top layer of tB-hBN with *θ* = 1.0° from the theoretical model and atomistic simulations. All scale bars are 10 nm. (h) Variations in the stress components (*σ_xx_, σ_zz_* and *σ_xz_*) at tB-G along the dashed lines shown in (e–g).

The fields of in-plane stress components (*σ_xx_, σ_zz_* and *σ_xz_*) in tB-G and tB-hBN with *θ* = 1.0° are shown in Fig. 4a–c and e–g, respectively. Similar to strain fields, the elevated stress was localized near the dislocation lines. We extracted the stress components along the dashed lines in Fig. 4a–c and e–g and plotted their variations in Fig. 4d and h, respectively. All the stress components reach their maximum absolute values at the dislocation lines (SP stacking) and then gradually decay to zero near the centers of the AB/BA stacking regions. We also investigated the out-of-plane stress components (*σ_yy_, σ_yz_* and *σ_xy_*) in the top layers of tB-G and tB-hBN with *θ* = 1.0° (Figs S7 and S8). Despite the slight increase in simulated values attributed to out-of-plane deformations, the distributions of these stress components align with theoretical predictions. Figure S9 shows the contours of in-plane stress components (*σ_xx_, σ_zz_* and *σ_xz_*) in tT-G with *θ* = 1.0°. Notably, the theoretical predictions show excellent agreement with the results from atomistic simulations.

### Energetic features of twisted 2D materials

**Figure 5.**
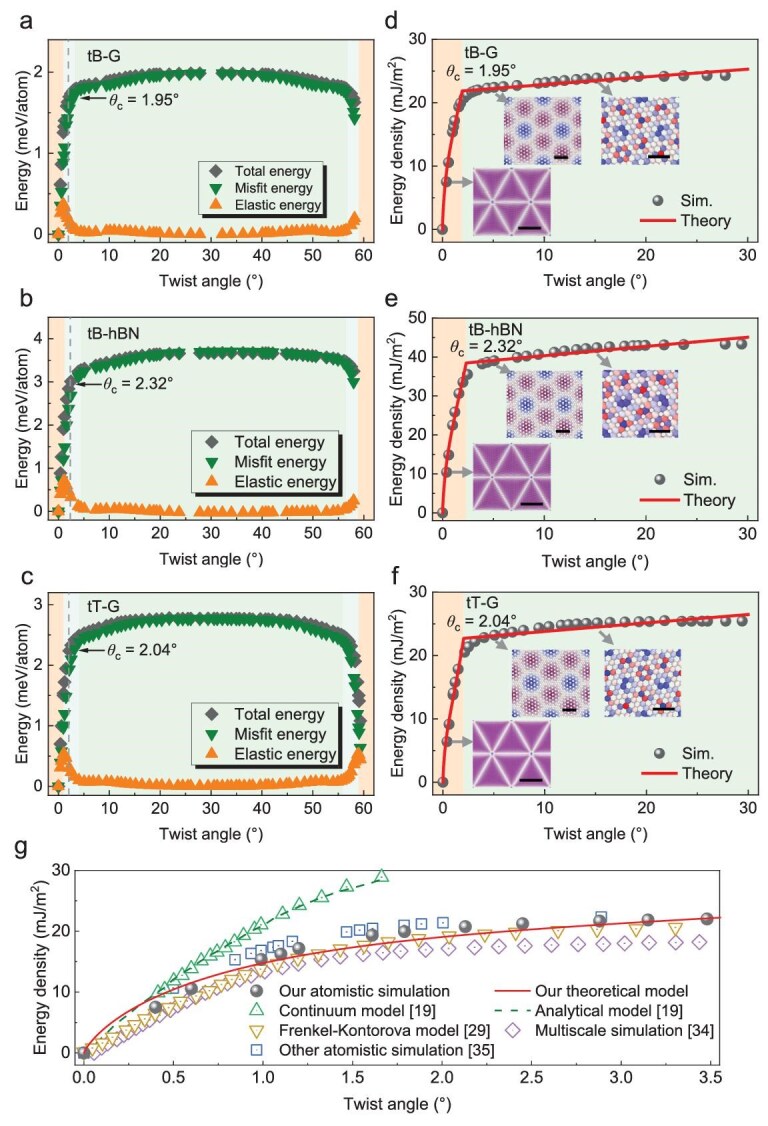
Energetic features of tB-G, tB-hBN and tT-G. (a–c) Total energy, interlayer misfit energy and intralayer elastic energy per atom for tB-G, tB-hBN and tT-G as functions of the twist angle from atomistic simulations. (d–f) Energy density for tB-G, tB-hBN and tT-G as functions of the twist angle from the theoretical model and atomistic simulations. Insets show the maps of the lateral distance *δ* for tB-G, tB-hBN and tT-G with *θ* = 0.4°, 5.1° and 15.2°, respectively. The scale bars in the insets with *θ* = 0.4°, 5.1° and 15.2° are 20, 1 and 0.5 nm, respectively. (g) Comparison of the energy density for tB-G with varying twist angles among our theoretical model, our atomistic simulations and previous studies [19,29,34,35].

Figure 5a illustrates the dependence of total energy, interlayer misfit energy and intralayer elastic energy per atom of tB-G on the twist angle from atomistic simulations. Owing to the hexagonal symmetry of monolayer graphene, the energy profiles exhibit mirror symmetry at *θ* = 30°. At small twist angles (*θ* < *θ_c_*), atomic-scale reconstruction results in in-plane deformation, significantly reducing the interlayer misfit energy of the overall system. As the twist angle increases, both the misfit energy and elastic energy rapidly increase and have comparable magnitudes. At large twist angles (*θ* > *θ_c_*), atomic-scale reconstruction becomes negligible, and in-plane deformation gradually weakens. The interlayer misfit energy is predominantly governed by the initial rotation and slowly increases with increasing *θ*, exhibiting a weak dependence on *θ*. The weakening of in-plane deformation leads to the nearly complete vanishing of elastic energy. Notably, for *θ* ranging from 0° to 30°, the misfit energy is always greater than the elastic energy, especially significantly exceeding the elastic energy at *θ* > *θ_c_*. As a result, the total energy exhibits a similar trend as the misfit energy. The total energy first increases sharply and then gradually saturates as the twist angle increases from 0° to 30°. The variations in all three energies of tB-hBN and tT-G are very similar to those of tB-G (Fig. 5b and c). The energy profiles for tB-hBN exhibit approximate mirror symmetry at 30°, which is attributed to the presence of two different atoms in hBN, breaking the inherent 6-fold symmetry.

Figure 5d–f presents the energy density (defined as the total energy divided by the interfacial area) of tB-G, tB-hBN and tT-G from our atomistic simulations. The peak energy densities of tB-G, tB-hBN and tT-G reach 24.3, 43.3 and 25.4 mJ/m^2^, respectively, and occur at *θ* = 30°. According to the deformation features and energy evolution shown in Fig. 5a–c, we separated the twist regime (0° < *θ* < 30°) into small-twist and large-twist regimens by introducing a critical twist angle *θ*_c_ [see Equation (10)]. By using Equation (10) to fit the energy density profile from atomistic simulations, we obtained the values of parameters *r*_0_, *E*_c_, *E*_dw_, *γ*_AA_ and *θ*_c_, which are listed in Table 1. The values of *θ*_c_ for tB-G, tB-hBN and tT-G are 1.95°, 2.32° and 2.04°, respectively. Notably, the values of *θ*_c_ for tB-G and tB-hBN are close to the critical *θ* at *d*_AA_ = *λ*/2 (shown in Fig. 2e and f), indicating that the energy of the AA-nodes cannot be ignored as their size approaches half of the superlattice periodicity. At small twist angles (*θ* < *θ*_c_), a triangular screw dislocation network is formed with atomic-scale reconstruction to reduce the interlayer misfit energy at the expense of intralayer elastic energy. As the twist angle increases, the triangular dislocation network becomes denser, leading to a rapid increase in total energy. At large twist angles (*θ* > *θ*_c_), as atomic-scale reconstruction nearly disappears, the interlayer misfit energy becomes a dominant factor in total energy, which is primarily determined by domains and domain walls.

**Table 1.** Fitting parameters for Equation (10) used for tB-G, tB-hBN and tT-G.

Parameter	tB-G	tB-hBN	tT-G
*r*_0_/*a*	0.53	0.70	0.85
${{{E}_{\mathrm{c}}} \mathord{/ {\vphantom {{{E}_{\mathrm{c}}} {{K}_{\mathrm{s}}b_{\mathrm{s}}^{\mathrm{2}}}}} } {{K}_{\mathrm{s}}b_{\mathrm{s}}^{\mathrm{2}}}}$	0.021	0.025	0.024
${{{E}_{{\mathrm{dw}}}} \mathord{/ {\vphantom {{{E}_{{\mathrm{dw}}}} {{K}_{\mathrm{s}}b_{\mathrm{s}}^{\mathrm{2}}}}} } {{K}_{\mathrm{s}}b_{\mathrm{s}}^{\mathrm{2}}}}$	0.00028	0.00041	0.00030
*γ*_AA_ (mJ/m^2^)	64.86	113.73	67.32
*θ*_c_ (°)	1.95	2.32	2.04

To validate the accuracy of our theoretical model, we compared the energy density of tB-G from our model with those from previous theoretical and computational studies [19,29,34,35], as shown in Fig. 5g. Nearly all previous studies focused on tB-G with small twist angles. Therefore, the comparison is limited within the regime of 0° < *θ* < 3.5°. Our theoretical predictions are in excellent agreement with results from previous studies, including atomistic simulations [35], multiscale simulations [34] and the Frenkel‒Kontorova model [29]. In the multiscale simulations, a non-linear finite element plate model was used to describe the mechanical response of each layer, and a discrete-continuum interlayer potential was used to describe the interlayer interactions [34]. In the Frenkel‒Kontorova model, the Föppl‒von Karman equations were used to calculate the elastic energy of each layer, and a generalized stacking‒fault energy (GSFE) was employed to determine the interlayer interaction energy [29]. Both multiscale simulations and the Frenkel‒Kontorova model were solved numerically, and there was no analytical solution for the deformation fields of twisted bilayer systems. Notably, there is a pronounced discrepancy between our theoretical predictions and the results from previous continuum and analytical models [19] at *θ* > 0.5°. In the continuum model, the elastic energy is described by the linear elastic thin plate theory, whereas the misfit energy is calculated via GSFE [19]. The equilibrium structure in the continuum model was determined by minimizing the total energy (a summation of misfit and elastic energy) via a semi-implicit numerical scheme [19]. The observed differences between our theoretical predictions and the results from the continuum model are attributed to the oversimplification of linear elastic plate theory in capturing the complex deformation induced by the atomic-scale reconstruction of tB-G at small twist angles. In the analytical model, the total energy of each primitive Moiré superlattice was considered as a summation of the energy of a single AA-node and the self-energy of three partial dislocation lines with a pure screw feature [19]. The assumption is that both the size and energy of the AA-node, as well as the energy per unit length of dislocation lines, remain constant. This model neglects the interactions between dislocation lines, leading to a discrepancy between our theoretical predictions and previous analytical models [19]. Compared with the results of previous studies [19,29,34,35], our theoretical model provides completely analytical expressions for the rotation, strain and stress fields and the energy density, and could be applicable to few-layer graphene and hBN across the small and large twist regimes.

## CONCLUSION

In summary, we have established a theoretical model to characterize the structural deformation and energetic features of twisted 2D materials (including tB-G, tB-hBN and tT-G) with varying twist angles. At a small twist angle (*θ* < *θ_c_*), the Moiré pattern of twisted 2D materials is dominated by triangular networks of partial dislocations, which are generated because of atomic-scale reconstruction. Our model provides full analytical expressions for the local rotation, strain and stress fields and describes the non-linear dependence of the energy density on the twist angle based on the anisotropic dislocation theory. At a large twist angle (*θ* > *θ_c_*), the Moiré superlattice consists of hexagonal domains with AA stacking and domain walls. Our model provides an analytical expression for the relationship between the energy density and twist angle. For 0° < *θ* <30°, the energy density of twisted 2D materials initially increases sharply and then plateaus as the twist angle increases. Furthermore, we have performed a series of large-scale atomistic simulations for tB-G, tB-hBN and tT-G to further validate and complement our theoretical model, and compared the results from our theoretical model with those from previous studies. Our theoretical predictions on the local rotation, strain and stress fields and the variation in energy density with twist angle are in good agreement with those from our atomistic simulations and previous experimental or computational studies. These findings indicate that our theoretical model can capture the structural deformation and energetic features of twisted 2D materials. The current model is applicable to various 2D materials ranging from single-element crystals (graphene) to binary compounds (hBN) and can be extended to twisted multilayer systems. The current work not only provides a fundamental understanding of the Moiré superlattice of twisted 2D materials but also establishes a comprehensive analytical framework for characterizing the deformation and energetic behaviors of twisted 2D materials.

## METHODS

### Atomistic simulations

To investigate the structural and energetic features of twisted 2D materials, we first constructed atomic models of tB-G, tB-hBN and tT-G with twist angles *θ *varying from 0° to 60°, according to the geometrical relationships of the top and bottom supercells in the twisted 2D bilayer [37]. We further performed a series of large-scale atomistic simulations for tB-G, tB-hBN and tT-G. During the simulations, we used the second-generation reactive empirical bond order (REBO) potential [38] and Kolmogorov‒Crespi (KC) potential [39,40] to describe the bonded intralayer and non-bonded interlayer interactions of C atoms in tB-G and tT-G, respectively. For the tB-hBN system, we employed the Tersoff-type empirical interatomic potential [41,42] and interlayer potential (ILP) [40] to describe the bonded intralayer and non-bonded interlayer interactions of B and N atoms, respectively. All simulations were performed via the large-scale atomic/molecular massively parallel simulator (LAMMPS) [43]. More details about the construction of atomic models and atomistic simulations are shown in Sections S2.1 and S2.2. For clarity, a schematic of the supercell construction is presented in Fig. S10, with the corresponding parameters summarized in Table S2.

## Supplementary Material

nwaf577_Supplemental_File
