# Supplementary material for: Strain, stress and rotation fields, and energetic features of twisted 2D materials
Source: Natl Sci Rev. 2025 Dec 16;13(4):nwaf577. doi: 10.1093/nsr/nwaf577 (PMC12883992; doi:10.1093/nsr/nwaf577)
Supplement: nwaf577_Supplemental_File [file nwaf577_supplemental_file.pdf]

Supplementary Material for

Strain, stress, and rotation fields, and energetic features of twisted 2D materials

Shuchang Li[1], Qian Zhang[1], Hanzheng Xing[1], Yuede Cao[1], Songyan Zhang[1], Xuan Zhang[1], Bin Ding[2], Xiaoyan Li[1,*]

[1]Mechano-X Institute, Applied Mechanics Laboratory, Department of Engineering Mechanics, Tsinghua University, Beijing 100084, China

[2]National Key Laboratory of Strength and Structural Integrity, Institute of Solid Mechanics, Beihang University, Beijing, 100191, China

[*]**Corresponding author.** Email: xiaoyanlithu@tsinghua.edu.cn.

This supplementary material contains the following sections:

1. Theoretical model
   - 1.1 Derivation of analytical expressions for local rotation, strain, and stress fields in twisted bilayer systems with the small twist angle
   - 1.2 Derivation of analytical expressions for energy density in twisted bilayer systems within both small and large twist regimes
   - 1.3 Derivation of analytical expressions for the local rotation, strain, stress fields, and energy density in twisted trilayer systems.

2. Atomistic simulations
   - 2.1 Construction of atomic models of tB-G, tB-hBN, and tT-G
   - 2.2 Atomistic simulation details

References

1. Theoretical model

1.1 Derivation of analytical expressions for local rotation, strain, and stress fields in twisted bilayer systems with the small twist angle

When a screw dislocation line is aligned along the z axis within a right-handed Cartesian coordinate system (x, y, z), three components (u_x, u_y and u_z) of its displacement field are given by,

$$
\begin{aligned}
u_x &= u_y = 0 \\
u_z &= -\frac{b_s}{2\pi}\arctan\left(\frac{\eta y}{x}\right)
\end{aligned}
\tag{S1}
$$

where b_s is the Burgers vector of partial dislocation and is equal to $a/\sqrt{3}$ (a is the lattice constant), and η is the anisotropy parameter related to the elastic constants of materials and its expression is provided subsequently.

As shown in Fig. 1i, at small twist angles, atomic-scale reconstruction in twisted bilayer systems induces the formation of a triangular dislocation network comprising three dislocation groups. Each group consists of an array of infinite parallel screw dislocations, uniformly spaced by a distance of D. By superimposing the displacement fields of the individual dislocations within the array, we obtained the following displacement field for the Group I dislocation array,

$$
\begin{aligned}
u_x^{\mathrm{I}} &= u_y^{\mathrm{I}} = 0 \\
u_z^{\mathrm{I}} &= -\frac{b_s}{2\pi}\sum_{n=-\infty}^{\infty}\arctan\left(\frac{\eta y}{x+nD}\right)
\end{aligned}
\tag{S2}
$$

The dislocation lines in Group I, II and III are oriented 60° apart. Consequently, we derived the displacement fields of Group II by rotating those of Group I by 60° counterclockwise, which are given as follows,

$$
\begin{aligned}
u_x^{\mathrm{II}} &= -\frac{\sqrt{3}b_s}{4\pi}\sum_{n=-\infty}^{\infty}\arctan\left(\frac{\eta y}{\left(\frac{1}{2}x-\frac{\sqrt{3}}{2}z\right)+nD}\right) \\
u_y^{\mathrm{II}} &= 0 \\
u_z^{\mathrm{II}} &= -\frac{b_s}{4\pi}\sum_{n=-\infty}^{\infty}\arctan\left(\frac{\eta y}{\left(\frac{1}{2}x-\frac{\sqrt{3}}{2}z\right)+nD}\right)
\end{aligned}
\tag{S3}
$$

Accordingly, we derived the displacement fields of Group III by rotating those of Group I by 60° clockwise, as presented below,

$$
\begin{aligned}
u_x^{\mathrm{III}} &= \frac{\sqrt{3}b_s}{4\pi}\sum_{n=-\infty}^{\infty}\arctan\left(\frac{\eta y}{\left(\frac{1}{2}x+\frac{\sqrt{3}}{2}z\right)+nD}\right) \\
u_y^{\mathrm{III}} &= 0
\end{aligned}
$$

$$u_z^{\mathrm{III}} = -\frac{b_s}{4\pi}\sum_{n=-\infty}^{\infty}\arctan\left(\frac{\eta y}{\left(\frac{1}{2}x+\frac{\sqrt{3}}{2}z\right)+nD}\right) \tag{S4}$$

By applying the principle of linear superposition to the displacement fields of three aforementioned groups, we further obtained the following displacement fields of total triangular dislocation network:

$$u_x = -\frac{\sqrt{3}b_s}{4\pi}\sum_{n=-\infty}^{\infty}\arctan\left(\frac{\eta y}{\left(\frac{1}{2}x-\frac{\sqrt{3}}{2}z\right)+nD}\right)-\arctan\left(\frac{\eta y}{\left(\frac{1}{2}x+\frac{\sqrt{3}}{2}z\right)+nD}\right)$$

$$u_y = 0 \tag{S5}$$

$$u_z = -\frac{b_s}{2\pi}\sum_{n=-\infty}^{\infty}\arctan\left(\frac{\eta y}{x+nD}\right)+\frac{1}{2}\arctan\left(\frac{\eta y}{\left(\frac{1}{2}x-\frac{\sqrt{3}}{2}z\right)+nD}\right)+\frac{1}{2}\arctan\left(\frac{\eta y}{\left(\frac{1}{2}x+\frac{\sqrt{3}}{2}z\right)+nD}\right)$$

To derive the expressions of the partial derivatives of the displacement components, we used the summation of infinite series as given in [1]. The basic equation is given by,

$$\sum_{m=-\infty}^{\infty}\frac{1}{m+l} = \pi\cot\pi l \tag{S6}$$

By summing the two expressions given by Eq. (S6) for $l=k+it$ and $l=k-it$, one obtains

$$\sum_{m=-\infty}^{\infty}\frac{m+k}{t^2+(m+k)^2} = \frac{\pi\sin 2\pi k}{\cosh 2\pi t-\cos 2\pi k} \tag{S7}$$

Subtracting the two expressions yields

$$\sum_{m=-\infty}^{\infty}\frac{1}{t^2+(m+k)^2} = \frac{\pi}{t}\frac{\sinh 2\pi t}{\cosh 2\pi t-\cos 2\pi k} \tag{S8}$$

By using Eqs. (S7) and (S8), we can obtain the analytical expressions of the partial derivatives of displacement components. Taking $\partial u_x/\partial x$ as an example, we can obtain the following expression,

$$\frac{\partial u_x}{\partial x} = -\frac{\sqrt{3}b_s}{4\pi}\sum_{n=-\infty}^{\infty}-\frac{1}{2}\frac{\eta y}{D^2}\frac{1}{\left(\frac{\eta y}{D}\right)^2+\left(\frac{\frac{1}{2}x-\frac{\sqrt{3}}{2}z}{D}+n\right)^2}-\frac{\sqrt{3}b_s}{4\pi}\sum_{n=-\infty}^{\infty}\frac{1}{2}\frac{\eta y}{D^2}\frac{1}{\left(\frac{\eta y}{D}\right)^2+\left(\frac{-\frac{1}{2}x-\frac{\sqrt{3}}{2}z}{D}+n\right)^2} \tag{S9}$$

By substituting $\left(\frac{1}{2}x-\frac{\sqrt{3}}{2}z\right)\Big/D = k_1$, $\eta y/D = t$, $\left(-\frac{1}{2}x-\frac{\sqrt{3}}{2}z\right)\Big/D = k_2$ and $\eta y/D = t$ into Eq. (S9), and then applying the summation of the infinite series from Eq. (S8), we further obtained the following expression,

$$\frac{\partial u_x}{\partial x} = \frac{\sqrt{3}b_s}{8D}\left\{\frac{\sinh(2\pi t)}{\cosh(2\pi t)-\cos(2\pi k_1)}-\frac{\sinh(2\pi t)}{\cosh(2\pi t)-\cos(2\pi k_2)}\right\} \tag{S10}$$

Thus, we write $\partial u_x/\partial x$ as follows

$$\frac{\partial u_x}{\partial x}=\frac{\sqrt{3}b_s}{8D}\left\{\frac{\sinh\left(2\pi\frac{\eta y}{D}\right)}{\cosh\left(2\pi\frac{\eta y}{D}\right)-\cos\left(\pi\frac{x-\sqrt{3}z}{D}\right)}-\frac{\sinh\left(2\pi\frac{\eta y}{D}\right)}{\cosh\left(2\pi\frac{\eta y}{D}\right)-\cos\left(\pi\frac{x+\sqrt{3}z}{D}\right)}\right\} \tag{S11}$$

Using the same procedure, we can obtain the other eight partial derivatives of the displacement components, as expressed below.

$$\frac{\partial u_x}{\partial y}=-\frac{\sqrt{3}b_s}{4D}\cdot\eta\left\{\frac{\sin\left(\pi\frac{x-\sqrt{3}z}{D}\right)}{\cosh\left(2\pi\frac{\eta y}{D}\right)-\cos\left(\pi\frac{x-\sqrt{3}z}{D}\right)}-\frac{\sin\left(\pi\frac{x+\sqrt{3}z}{D}\right)}{\cosh\left(2\pi\frac{\eta y}{D}\right)-\cos\left(\pi\frac{x+\sqrt{3}z}{D}\right)}\right\}$$

$$\frac{\partial u_x}{\partial z}=-\frac{3b_s}{8D}\left\{\frac{\sinh\left(2\pi\frac{\eta y}{D}\right)}{\cosh\left(2\pi\frac{\eta y}{D}\right)-\cos\left(\pi\frac{x-\sqrt{3}z}{D}\right)}+\frac{\sinh\left(2\pi\frac{\eta y}{D}\right)}{\cosh\left(2\pi\frac{\eta y}{D}\right)-\cos\left(\pi\frac{x+\sqrt{3}z}{D}\right)}\right\}$$

$$\frac{\partial u_y}{\partial x}=\frac{\partial u_y}{\partial y}=\frac{\partial u_y}{\partial z}=0$$

$$\frac{\partial u_z}{\partial x}=\frac{b_s}{2D}\left\{\frac{\sinh\left(2\pi\frac{\eta y}{D}\right)}{\cosh\left(2\pi\frac{\eta y}{D}\right)-\cos\left(2\pi\frac{x}{D}\right)}+\frac{1}{4}\frac{\sinh\left(2\pi\frac{\eta y}{D}\right)}{\cosh\left(2\pi\frac{\eta y}{D}\right)-\cos\left(\pi\frac{x-\sqrt{3}z}{D}\right)}+\frac{1}{4}\frac{\sinh\left(2\pi\frac{\eta y}{D}\right)}{\cosh\left(2\pi\frac{\eta y}{D}\right)-\cos\left(\pi\frac{x+\sqrt{3}z}{D}\right)}\right\}$$

$$\frac{\partial u_z}{\partial y}=-\frac{b_s}{2D}\cdot\eta\left\{\frac{\sin\left(2\pi\frac{x}{D}\right)}{\cosh\left(2\pi\frac{\eta y}{D}\right)-\cos\left(2\pi\frac{x}{D}\right)}+\frac{1}{2}\frac{\sin\left(\pi\frac{x-\sqrt{3}z}{D}\right)}{\cosh\left(2\pi\frac{\eta y}{D}\right)-\cos\left(\pi\frac{x-\sqrt{3}z}{D}\right)}+\frac{1}{2}\frac{\sin\left(\pi\frac{x+\sqrt{3}z}{D}\right)}{\cosh\left(2\pi\frac{\eta y}{D}\right)-\cos\left(\pi\frac{x+\sqrt{3}z}{D}\right)}\right\}$$

$$\frac{\partial u_z}{\partial z}=-\frac{\sqrt{3}b_s}{8D}\left\{\frac{\sinh\left(2\pi\frac{\eta y}{D}\right)}{\cosh\left(2\pi\frac{\eta y}{D}\right)-\cos\left(\pi\frac{x-\sqrt{3}z}{D}\right)}-\frac{\sinh\left(2\pi\frac{\eta y}{D}\right)}{\cosh\left(2\pi\frac{\eta y}{D}\right)-\cos\left(\pi\frac{x+\sqrt{3}z}{D}\right)}\right\}$$

(S12)

Based on the geometric equations and expressions in Eqs. (S11) and (S12), we obtained the following expression of total rotation field ϕ_T,

$$\phi_{\mathrm{T}}=\frac{1}{2}\left(\frac{\partial u_z}{\partial x}-\frac{\partial u_x}{\partial z}\right)=\frac{b_s}{4D}\left\{\begin{array}{l}\dfrac{\sinh\left(2\pi\dfrac{\eta y}{D}\right)}{\cosh\left(2\pi\dfrac{\eta y}{D}\right)-\cos\left(2\pi\dfrac{x}{D}\right)}+\dfrac{\sinh\left(2\pi\dfrac{\eta y}{D}\right)}{\cosh\left(2\pi\dfrac{\eta y}{D}\right)-\cos\left(\pi\dfrac{x-\sqrt{3}z}{D}\right)}\\+\dfrac{\sinh\left(2\pi\dfrac{\eta y}{D}\right)}{\cosh\left(2\pi\dfrac{\eta y}{D}\right)-\cos\left(\pi\dfrac{x+\sqrt{3}z}{D}\right)}\end{array}\right\}\quad\text{(S13)}$$

By eliminating the initial twist angle θ, we further obtained the following expression of local rotation angle ϕ_{R} of top layer relative to the bottom layer in twisted bilayer systems,

$$\phi_{\mathrm{R}}(x,z)=\phi_{\mathrm{T}}\left(x,\frac{h}{2},z\right)-\phi_{\mathrm{T}}\left(x,-\frac{h}{2},z\right)-\theta\quad\text{(S14)}$$

We also obtained the following expressions of strain fields based on the geometric equations and expressions in Eqs. (S11) and (S12),

$$\varepsilon_{xx}=\frac{\partial u_x}{\partial x}=\frac{\sqrt{3}b_s}{8D}\left\{\frac{\sinh\left(2\pi\dfrac{\eta y}{D}\right)}{\cosh\left(2\pi\dfrac{\eta y}{D}\right)-\cos\left(\pi\dfrac{x-\sqrt{3}z}{D}\right)}-\frac{\sinh\left(2\pi\dfrac{\eta y}{D}\right)}{\cosh\left(2\pi\dfrac{\eta y}{D}\right)-\cos\left(\pi\dfrac{x+\sqrt{3}z}{D}\right)}\right\}$$

$$\varepsilon_{yy}=0$$

$$\varepsilon_{zz}=\frac{\partial u_z}{\partial z}=-\frac{\sqrt{3}b_s}{8D}\left\{\frac{\sinh\left(2\pi\dfrac{\eta y}{D}\right)}{\cosh\left(2\pi\dfrac{\eta y}{D}\right)-\cos\left(\pi\dfrac{x-\sqrt{3}z}{D}\right)}-\frac{\sinh\left(2\pi\dfrac{\eta y}{D}\right)}{\cosh\left(2\pi\dfrac{\eta y}{D}\right)-\cos\left(\pi\dfrac{x+\sqrt{3}z}{D}\right)}\right\}$$

$$\varepsilon_{yz}=\frac{1}{2}\left(\frac{\partial u_y}{\partial z}+\frac{\partial u_z}{\partial y}\right)=-\frac{b_s}{4D}\cdot\eta\left\{\begin{array}{l}\dfrac{\sin\left(2\pi\dfrac{x}{D}\right)}{\cosh\left(2\pi\dfrac{\eta y}{D}\right)-\cos\left(2\pi\dfrac{x}{D}\right)}+\dfrac{1}{2}\dfrac{\sin\left(\pi\dfrac{x-\sqrt{3}z}{D}\right)}{\cosh\left(2\pi\dfrac{\eta y}{D}\right)-\cos\left(\pi\dfrac{x-\sqrt{3}z}{D}\right)}\\+\dfrac{1}{2}\dfrac{\sin\left(\pi\dfrac{x+\sqrt{3}z}{D}\right)}{\cosh\left(2\pi\dfrac{\eta y}{D}\right)-\cos\left(\pi\dfrac{x+\sqrt{3}z}{D}\right)}\end{array}\right\}$$

$$\varepsilon_{xz}=\frac{1}{2}\left(\frac{\partial u_x}{\partial z}+\frac{\partial u_z}{\partial x}\right)=\frac{b_s}{4D}\left\{\begin{array}{l}\dfrac{\sinh\left(2\pi\dfrac{\eta y}{D}\right)}{\cosh\left(2\pi\dfrac{\eta y}{D}\right)-\cos\left(2\pi\dfrac{x}{D}\right)}-\dfrac{1}{2}\dfrac{\sinh\left(2\pi\dfrac{\eta y}{D}\right)}{\cosh\left(2\pi\dfrac{\eta y}{D}\right)-\cos\left(\pi\dfrac{x-\sqrt{3}z}{D}\right)}\\-\dfrac{1}{2}\dfrac{\sinh\left(2\pi\dfrac{\eta y}{D}\right)}{\cosh\left(2\pi\dfrac{\eta y}{D}\right)-\cos\left(\pi\dfrac{x+\sqrt{3}z}{D}\right)}\end{array}\right\}$$

$$\varepsilon_{xy}=\frac{1}{2}\left(\frac{\partial u_x}{\partial y}+\frac{\partial u_y}{\partial x}\right)=-\frac{\sqrt{3}b_s}{8D}\cdot\eta\left\{\frac{\sin\left(\pi\frac{x-\sqrt{3}z}{D}\right)}{\cosh\left(2\pi\frac{\eta y}{D}\right)-\cos\left(\pi\frac{x-\sqrt{3}z}{D}\right)}-\frac{\sin\left(\pi\frac{x+\sqrt{3}z}{D}\right)}{\cosh\left(2\pi\frac{\eta y}{D}\right)-\cos\left(\pi\frac{x+\sqrt{3}z}{D}\right)}\right\}$$

(S15)

Due to their weak interlayer van der Waals (vdW) interactions and strong intralayer bonded interactions, two-dimensional (2D) materials can be regarded as transversely isotropic materials. Thus, the elastic constant matrix can be expressed as,

$$\boldsymbol{C}=\begin{pmatrix} C_{11} & C_{13} & C_{12} & 0 & 0 & 0 \\ C_{13} & C_{33} & C_{13} & 0 & 0 & 0 \\ C_{12} & C_{13} & C_{11} & 0 & 0 & 0 \\ 0 & 0 & 0 & C_{44} & 0 & 0 \\ 0 & 0 & 0 & 0 & \frac{C_{11}-C_{12}}{2} & 0 \\ 0 & 0 & 0 & 0 & 0 & C_{44} \end{pmatrix} \tag{S16}$$

For 2D materials, the anisotropy parameter η is defined as,

$$\eta^2=\frac{1}{2}\left(C_{11}-C_{12}\right)/C_{44} \tag{S17}$$

For linear elastic deformation of 2D materials, the relationship between stress $\boldsymbol{\sigma}=[\sigma_{xx}\ \sigma_{yy}\ \sigma_{zz}\ \sigma_{yz}\ \sigma_{xz}\ \sigma_{xy}]^T$ and strain $\boldsymbol{\varepsilon}=[\ \varepsilon_{xx}\ \varepsilon_{yy}\ \varepsilon_{zz}\ \varepsilon_{yz}\ \varepsilon_{xz}\ \varepsilon_{xy}]^T$ follows the generalized Hooke' s law,

$$\boldsymbol{\sigma}=\boldsymbol{C}\boldsymbol{\varepsilon} \tag{S18}$$

By substituting Eqs. (S15) and (S16) into Eq. (S18), we obtained the following expressions of stress fields,

$$\sigma_{xx}=\frac{\sqrt{3}b_s}{4D}\cdot K_s\eta\left\{\frac{\sinh\left(2\pi\frac{\eta y}{D}\right)}{\cosh\left(2\pi\frac{\eta y}{D}\right)-\cos\left(\pi\frac{x-\sqrt{3}z}{D}\right)}-\frac{\sinh\left(2\pi\frac{\eta y}{D}\right)}{\cosh\left(2\pi\frac{\eta y}{D}\right)-\cos\left(\pi\frac{x+\sqrt{3}z}{D}\right)}\right\}$$

$$\sigma_{yy}=0$$

$$\sigma_{zz}=-\frac{\sqrt{3}b_s}{4D}\cdot K_s\eta\left\{\frac{\sinh\left(2\pi\frac{\eta y}{D}\right)}{\cosh\left(2\pi\frac{\eta y}{D}\right)-\cos\left(\pi\frac{x-\sqrt{3}z}{D}\right)}-\frac{\sinh\left(2\pi\frac{\eta y}{D}\right)}{\cosh\left(2\pi\frac{\eta y}{D}\right)-\cos\left(\pi\frac{x+\sqrt{3}z}{D}\right)}\right\}$$

$$\sigma_{yz} = -\frac{b_s}{2D}\cdot K_s \left\{ \begin{array}{l} \dfrac{\sin\left(2\pi\dfrac{x}{D}\right)}{\cosh\left(2\pi\dfrac{\eta y}{D}\right)-\cos\left(2\pi\dfrac{x}{D}\right)} + \dfrac{1}{2}\dfrac{\sin\left(\pi\dfrac{x-\sqrt{3}z}{D}\right)}{\cosh\left(2\pi\dfrac{\eta y}{D}\right)-\cos\left(\pi\dfrac{x-\sqrt{3}z}{D}\right)} \\ +\dfrac{1}{2}\dfrac{\sin\left(\pi\dfrac{x+\sqrt{3}z}{D}\right)}{\cosh\left(2\pi\dfrac{\eta y}{D}\right)-\cos\left(\pi\dfrac{x+\sqrt{3}z}{D}\right)} \end{array} \right\}$$

$$\sigma_{xz} = \frac{b_s}{2D}\cdot K_s\eta \left\{ \begin{array}{l} \dfrac{\sinh\left(2\pi\dfrac{\eta y}{D}\right)}{\cosh\left(2\pi\dfrac{\eta y}{D}\right)-\cos\left(2\pi\dfrac{x}{D}\right)} - \dfrac{1}{2}\dfrac{\sinh\left(2\pi\dfrac{\eta y}{D}\right)}{\cosh\left(2\pi\dfrac{\eta y}{D}\right)-\cos\left(\pi\dfrac{x-\sqrt{3}z}{D}\right)} \\ -\dfrac{1}{2}\dfrac{\sinh\left(2\pi\dfrac{\eta y}{D}\right)}{\cosh\left(2\pi\dfrac{\eta y}{D}\right)-\cos\left(\pi\dfrac{x+\sqrt{3}z}{D}\right)} \end{array} \right\} \tag{S19}$$

$$\sigma_{xy} = -\frac{\sqrt{3}b_s}{4D}\cdot K_s \left\{ \frac{\sin\left(\pi\dfrac{x-\sqrt{3}z}{D}\right)}{\cosh\left(2\pi\dfrac{\eta y}{D}\right)-\cos\left(\pi\dfrac{x-\sqrt{3}z}{D}\right)} - \frac{\sin\left(\pi\dfrac{x+\sqrt{3}z}{D}\right)}{\cosh\left(2\pi\dfrac{\eta y}{D}\right)-\cos\left(\pi\dfrac{x+\sqrt{3}z}{D}\right)} \right\}$$

where K_s represents the energy coefficient and is defined as,

$$K_s = \left[\frac{1}{2}\left(C_{11}-C_{12}\right)C_{44}\right]^{\frac{1}{2}} \tag{S20}$$

By taking the partial derivative of Eq. (S15), we obtained the strain gradient fields, which are expressed as,

$$\varepsilon_{xx,x} = \frac{\sqrt{3}\pi b_s}{8D^2}\left\{ -\frac{\sin\left(\pi\dfrac{x-\sqrt{3}z}{D}\right)\sinh\left(2\pi\dfrac{\eta y}{D}\right)}{\left[\cosh\left(2\pi\dfrac{\eta y}{D}\right)-\cos\left(\pi\dfrac{x-\sqrt{3}z}{D}\right)\right]^2} + \frac{\sin\left(\pi\dfrac{x+\sqrt{3}z}{D}\right)\sinh\left(2\pi\dfrac{\eta y}{D}\right)}{\left[\cosh\left(2\pi\dfrac{\eta y}{D}\right)-\cos\left(\pi\dfrac{x+\sqrt{3}z}{D}\right)\right]^2} \right\}$$

$$\varepsilon_{xx,z} = \frac{3\pi b_s}{8D^2}\left\{ \frac{\sin\left(\pi\dfrac{x-\sqrt{3}z}{D}\right)\sinh\left(2\pi\dfrac{\eta y}{D}\right)}{\left[\cosh\left(2\pi\dfrac{\eta y}{D}\right)-\cos\left(\pi\dfrac{x-\sqrt{3}z}{D}\right)\right]^2} + \frac{\sin\left(\pi\dfrac{x+\sqrt{3}z}{D}\right)\sinh\left(2\pi\dfrac{\eta y}{D}\right)}{\left[\cosh\left(2\pi\dfrac{\eta y}{D}\right)-\cos\left(\pi\dfrac{x+\sqrt{3}z}{D}\right)\right]^2} \right\}$$

$$\varepsilon_{xx,y} = \frac{\sqrt{3}\pi b_s}{2D^2}\cdot\eta\left\{ \frac{1-\cos\left(\pi\dfrac{x-\sqrt{3}z}{D}\right)\cosh\left(2\pi\dfrac{\eta y}{D}\right)}{\left[\cosh\left(2\pi\dfrac{\eta y}{D}\right)-\cos\left(\pi\dfrac{x-\sqrt{3}z}{D}\right)\right]^2} - \frac{1-\cos\left(\pi\dfrac{x+\sqrt{3}z}{D}\right)\cosh\left(2\pi\dfrac{\eta y}{D}\right)}{\left[\cosh\left(2\pi\dfrac{\eta y}{D}\right)-\cos\left(\pi\dfrac{x+\sqrt{3}z}{D}\right)\right]^2} \right\}$$

$$\varepsilon_{zz,x}=-\frac{\sqrt{3}\pi b_s}{8D^2}\left\{-\frac{\sin\left(\pi\frac{x-\sqrt{3}z}{D}\right)\sinh\left(2\pi\frac{\eta y}{D}\right)}{\left[\cosh\left(2\pi\frac{\eta y}{D}\right)-\cos\left(\pi\frac{x-\sqrt{3}z}{D}\right)\right]^2}+\frac{\sin\left(\pi\frac{x+\sqrt{3}z}{D}\right)\sinh\left(2\pi\frac{\eta y}{D}\right)}{\left[\cosh\left(2\pi\frac{\eta y}{D}\right)-\cos\left(\pi\frac{x+\sqrt{3}z}{D}\right)\right]^2}\right\}$$

$$\varepsilon_{zz,z}=-\frac{3\pi b_s}{8D^2}\left\{\frac{\sin\left(\pi\frac{x-\sqrt{3}z}{D}\right)\sinh\left(2\pi\frac{\eta y}{D}\right)}{\left[\cosh\left(2\pi\frac{\eta y}{D}\right)-\cos\left(\pi\frac{x-\sqrt{3}z}{D}\right)\right]^2}+\frac{\sin\left(\pi\frac{x+\sqrt{3}z}{D}\right)\sinh\left(2\pi\frac{\eta y}{D}\right)}{\left[\cosh\left(2\pi\frac{\eta y}{D}\right)-\cos\left(\pi\frac{x+\sqrt{3}z}{D}\right)\right]^2}\right\}$$

$$\varepsilon_{zz,y}=-\frac{\sqrt{3}\pi b_s}{2D^2}\cdot\eta\left\{\frac{1-\cos\left(\pi\frac{x-\sqrt{3}z}{D}\right)\cosh\left(2\pi\frac{\eta y}{D}\right)}{\left[\cosh\left(2\pi\frac{\eta y}{D}\right)-\cos\left(\pi\frac{x-\sqrt{3}z}{D}\right)\right]^2}-\frac{1-\cos\left(\pi\frac{x+\sqrt{3}z}{D}\right)\cosh\left(2\pi\frac{\eta y}{D}\right)}{\left[\cosh\left(2\pi\frac{\eta y}{D}\right)-\cos\left(\pi\frac{x+\sqrt{3}z}{D}\right)\right]^2}\right\}$$

$$\varepsilon_{zx,x}=\frac{\pi b_s}{2D^2}\left\{\begin{array}{l}-\frac{\sinh\left(2\pi\frac{\eta y}{D}\right)\sin\left(2\pi\frac{x}{D}\right)}{\left[\cosh\left(2\pi\frac{\eta y}{D}\right)-\cos\left(2\pi\frac{x}{D}\right)\right]^2}+\frac{1}{4}\frac{\sinh\left(2\pi\frac{\eta y}{D}\right)\sin\left(\pi\frac{x-\sqrt{3}z}{D}\right)}{\left[\cosh\left(2\pi\frac{\eta y}{D}\right)-\cos\left(\pi\frac{x-\sqrt{3}z}{D}\right)\right]^2}\\+\frac{1}{4}\frac{\sinh\left(2\pi\frac{\eta y}{D}\right)\sin\left(\pi\frac{x+\sqrt{3}z}{D}\right)}{\left[\cosh\left(2\pi\frac{\eta y}{D}\right)-\cos\left(\pi\frac{x+\sqrt{3}z}{D}\right)\right]^2}\end{array}\right\}$$

$$\varepsilon_{zx,z}=\frac{\sqrt{3}\pi b_s}{8D^2}\left\{-\frac{\sinh\left(2\pi\frac{\eta y}{D}\right)\sin\left(\pi\frac{x-\sqrt{3}z}{D}\right)}{\left[\cosh\left(2\pi\frac{\eta y}{D}\right)-\cos\left(\pi\frac{x-\sqrt{3}z}{D}\right)\right]^2}+\frac{\sinh\left(2\pi\frac{\eta y}{D}\right)\sin\left(\pi\frac{x+\sqrt{3}z}{D}\right)}{\left[\cosh\left(2\pi\frac{\eta y}{D}\right)-\cos\left(\pi\frac{x+\sqrt{3}z}{D}\right)\right]^2}\right\}$$

$$\varepsilon_{zx,y}=\frac{\pi b_s}{2D^2}\cdot\eta\left\{\begin{array}{l}\frac{1-\cosh\left(2\pi\frac{\eta y}{D}\right)\cos\left(2\pi\frac{x}{D}\right)}{\left[\cosh\left(2\pi\frac{\eta y}{D}\right)-\cos\left(2\pi\frac{x}{D}\right)\right]^2}-\frac{1}{2}\frac{1-\cos\left(\pi\frac{x-\sqrt{3}z}{D}\right)\cosh\left(2\pi\frac{\eta y}{D}\right)}{\left[\cosh\left(2\pi\frac{\eta y}{D}\right)-\cos\left(\pi\frac{x-\sqrt{3}z}{D}\right)\right]^2}\\-\frac{1}{2}\frac{1-\cos\left(\pi\frac{x+\sqrt{3}z}{D}\right)\cosh\left(2\pi\frac{\eta y}{D}\right)}{\left[\cosh\left(2\pi\frac{\eta y}{D}\right)-\cos\left(\pi\frac{x+\sqrt{3}z}{D}\right)\right]^2}\end{array}\right\}$$

$$\varepsilon_{yz,x}=-\frac{\pi b_s}{2D^2}\cdot\eta\left\{\begin{array}{l}\dfrac{\cosh\left(2\pi\dfrac{\eta y}{D}\right)\cos\left(2\pi\dfrac{x}{D}\right)-1}{\left[\cosh\left(2\pi\dfrac{\eta y}{D}\right)-\cos\left(2\pi\dfrac{x}{D}\right)\right]^2}-\dfrac{1}{4}\dfrac{\cos\left(\pi\dfrac{x-\sqrt{3}z}{D}\right)\cosh\left(2\pi\dfrac{\eta y}{D}\right)-1}{\left[\cosh\left(2\pi\dfrac{\eta y}{D}\right)-\cos\left(\pi\dfrac{x-\sqrt{3}z}{D}\right)\right]^2}\\-\dfrac{1}{4}\dfrac{\cos\left(\pi\dfrac{x+\sqrt{3}z}{D}\right)\cosh\left(2\pi\dfrac{\eta y}{D}\right)-1}{\left[\cosh\left(2\pi\dfrac{\eta y}{D}\right)-\cos\left(\pi\dfrac{x+\sqrt{3}z}{D}\right)\right]^2}\end{array}\right\}$$

$$\varepsilon_{yz,z}=-\frac{\sqrt{3}\pi b_s}{8D^2}\cdot\eta\left\{-\frac{\cos\left(\pi\dfrac{x-\sqrt{3}z}{D}\right)\cosh\left(2\pi\dfrac{\eta y}{D}\right)-1}{\left[\cosh\left(2\pi\dfrac{\eta y}{D}\right)-\cos\left(\pi\dfrac{x-\sqrt{3}z}{D}\right)\right]^2}+\frac{\cos\left(\pi\dfrac{x+\sqrt{3}z}{D}\right)\cosh\left(2\pi\dfrac{\eta y}{D}\right)-1}{\left[\cosh\left(2\pi\dfrac{\eta y}{D}\right)-\cos\left(\pi\dfrac{x+\sqrt{3}z}{D}\right)\right]^2}\right\}$$

$$\varepsilon_{yz,y}=-\frac{\pi b_s}{2D^2}\cdot\eta^2\left\{\begin{array}{l}-\dfrac{\sinh\left(2\pi\dfrac{\eta y}{D}\right)\sin\left(2\pi\dfrac{x}{D}\right)}{\left[\cosh\left(2\pi\dfrac{\eta y}{D}\right)-\cos\left(2\pi\dfrac{x}{D}\right)\right]^2}-\dfrac{1}{2}\dfrac{\sinh\left(2\pi\dfrac{\eta y}{D}\right)\sin\left(\pi\dfrac{x-\sqrt{3}z}{D}\right)}{\left[\cosh\left(2\pi\dfrac{\eta y}{D}\right)-\cos\left(\pi\dfrac{x-\sqrt{3}z}{D}\right)\right]^2}\\-\dfrac{1}{2}\dfrac{\sinh\left(2\pi\dfrac{\eta y}{D}\right)\sin\left(\pi\dfrac{x+\sqrt{3}z}{D}\right)}{\left[\cosh\left(2\pi\dfrac{\eta y}{D}\right)-\cos\left(\pi\dfrac{x+\sqrt{3}z}{D}\right)\right]^2}\end{array}\right\}$$

$$\varepsilon_{xy,x}=-\frac{\sqrt{3}\pi b_s}{8D^2}\cdot\eta\left\{\frac{\cos\left(\pi\dfrac{x-\sqrt{3}z}{D}\right)\cosh\left(2\pi\dfrac{\eta y}{D}\right)-1}{\left[\cosh\left(2\pi\dfrac{\eta y}{D}\right)-\cos\left(\pi\dfrac{x-\sqrt{3}z}{D}\right)\right]^2}-\frac{\cos\left(\pi\dfrac{x+\sqrt{3}z}{D}\right)\cosh\left(2\pi\dfrac{\eta y}{D}\right)-1}{\left[\cosh\left(2\pi\dfrac{\eta y}{D}\right)-\cos\left(\pi\dfrac{x+\sqrt{3}z}{D}\right)\right]^2}\right\}$$

$$\varepsilon_{xy,z}=-\frac{3\pi b_s}{8D^2}\cdot\eta\left\{-\frac{\cos\left(\pi\dfrac{x-\sqrt{3}z}{D}\right)\cosh\left(2\pi\dfrac{\eta y}{D}\right)-1}{\left[\cosh\left(2\pi\dfrac{\eta y}{D}\right)-\cos\left(\pi\dfrac{x-\sqrt{3}z}{D}\right)\right]^2}-\frac{\cos\left(\pi\dfrac{x+\sqrt{3}z}{D}\right)\cosh\left(2\pi\dfrac{\eta y}{D}\right)-1}{\left[\cosh\left(2\pi\dfrac{\eta y}{D}\right)-\cos\left(\pi\dfrac{x+\sqrt{3}z}{D}\right)\right]^2}\right\}$$

$$\varepsilon_{xy,y}=-\frac{\sqrt{3}\pi b_s}{4D^2}\cdot\eta^2\left\{-\frac{\sinh\left(2\pi\dfrac{\eta y}{D}\right)\sin\left(\pi\dfrac{x-\sqrt{3}z}{D}\right)}{\left[\cosh\left(2\pi\dfrac{\eta y}{D}\right)-\cos\left(\pi\dfrac{x-\sqrt{3}z}{D}\right)\right]^2}+\frac{\sinh\left(2\pi\dfrac{\eta y}{D}\right)\sin\left(\pi\dfrac{x+\sqrt{3}z}{D}\right)}{\left[\cosh\left(2\pi\dfrac{\eta y}{D}\right)-\cos\left(\pi\dfrac{x+\sqrt{3}z}{D}\right)\right]^2}\right\}$$

(S21)

1.2 Derivation of analytical expressions for energy density in twisted bilayer systems within both small and large twist regimes

At small twist angles ($\theta<\theta_c$, where θ_c is a critical twist angle), atomic-scale reconstruction in a bilayer 2D material induces the formation of a triangular network of partial screw dislocations at the twist interface. To evaluate the energy of the twist interface, we first considered a pair of triangular dislocation networks with opposite signs embedded in an infinite crystal. The specific energy required to separate such a pair of dislocation networks is equal to twice the elastic energy of the twist interface per unit area. The attractive force (per unit length) of the dislocation of opposite sign along the out-of-plane direction is equivalent to -$\sigma_{xz}b_s$. The elastic energy W_{el} per Moiré superlattice period λ per dislocation of the twist interface is given by,

$$W_{\mathrm{el}} = \frac{1}{2}\int_0^{\lambda}\int_{r_0}^{\infty}\sigma_{xz}\cdot b_s \mathrm{d}y\mathrm{d}z \tag{S22}$$

where r_0 is the core radius of dislocation. By substituting Eq. (S19) into Eq. (S22), we obtained the following expression of elastic energy,

$$W_{\mathrm{el}} = \int_0^{\lambda}\int_{r_0}^{\infty}\frac{K_s b_s^2}{4D}\cdot\eta\cdot\left\{\frac{\sinh\left(2\pi\frac{\eta y}{D}\right)}{\cosh\left(2\pi\frac{\eta y}{D}\right)-1}-\frac{\sinh\left(2\pi\frac{\eta y}{D}\right)}{\cosh\left(2\pi\frac{\eta y}{D}\right)-\cos\left(\frac{\sqrt{3}\pi}{D}z\right)}\right\}\mathrm{d}y\mathrm{d}z \tag{S23}$$

By applying the variable substitution with $\psi = 2\pi\eta y/D$ and $\zeta = \sqrt{3}\pi z/D$, we evaluated the integral with respect to y in Eq. (S27) as presented in the following expressions [2],

$$\begin{aligned}
&\int_{r_0}^{\infty}\frac{K_s b_s^2}{4D}\cdot\eta\cdot\left\{\frac{\sinh\left(2\pi\frac{\eta y}{D}\right)}{\cosh\left(2\pi\frac{\eta y}{D}\right)-1}-\frac{\sinh\left(2\pi\frac{\eta y}{D}\right)}{\cosh\left(2\pi\frac{\eta y}{D}\right)-\cos\left(\frac{\sqrt{3}\pi}{D}z\right)}\right\}\mathrm{d}y \\
&=\frac{K_s b_s^2}{8\pi}\int_{\psi_0}^{\infty}\left\{\frac{\sinh\psi}{\cosh\psi-1}-\frac{\sinh\psi}{\cosh\psi-\cos\zeta}\right\}\mathrm{d}\psi \\
&=\frac{K_s b_s^2}{8\pi}\cdot\left[\ln\left(\cosh\psi_0-\cos\zeta\right)-\ln\left(\cos\psi_0-1\right)\right]
\end{aligned} \tag{S24}$$

where $\psi_0 = 2\pi\eta r_0/D$. The expression of W_{el} is further written as,

$$\begin{aligned}
W_{\mathrm{el}} &= \frac{K_s b_s^2}{8\pi}\cdot\frac{D}{\sqrt{3}\pi}\int_0^{2\pi}\left[\ln\left(\cosh\psi_0-\cos\zeta\right)-\ln\left(\cos\psi_0-1\right)\right]\mathrm{d}\zeta \\
&=\frac{K_s b_s^2 D}{4\sqrt{3}\pi}\left\{\ln\left[\cosh\psi_0+\left(\cosh^2\psi_0-1\right)^{\frac{1}{2}}\right]-\ln\left(\cosh\psi_0-1\right)-\ln 2\right\} \\
&=\frac{K_s b_s^2 D}{4\sqrt{3}\pi}\left\{\psi_0-2\ln\left[\sinh\left(\frac{\psi_0}{2}\right)\right]-2\ln 2\right\}
\end{aligned} \tag{S25}$$

Substituting $\psi_0 = 2\pi\eta r_0/D$ into Eq. (S25), we obtained the following expression,

$$W_{\mathrm{el}}=\frac{K_s b_s^2 D}{2\sqrt{3}\pi}\left\{\frac{\pi\eta}{D}r_0-\ln\left[\sinh\left(\frac{\pi\eta}{D}r_0\right)\right]-\ln 2\right\} \tag{S26}$$

Consequently, we calculated the elastic energy density γ_{el} (elastic energy per unit area) of twisted bilayer systems, which is expressed as

$$\gamma_{\mathrm{el}}=\frac{3\sqrt{3}W_{\mathrm{el}}}{2D^2} \tag{S27}$$

In addition to the elastic energy, we also took the core energy of dislocation into account. Assuming a constant core energy per unit length of dislocation line, we determined the following core energy density γ_{c} (core energy per unit area),

$$\gamma_{\mathrm{c}}=3\frac{E_{\mathrm{c}}}{D} \tag{S28}$$

where E_{c} represents the core energy per unit length of dislocation line. By summing the elastic and core energies of dislocation network, we determined the total energy of the twist interface featuring a triangular network of partial screw dislocations in a bilayer 2D material. Given that $D=\sqrt{3}a/2\theta$ and $b_{\mathrm{s}}=a/\sqrt{3}$, we finally formulated the energy density γ_{twist} at small twist angles as,

$$\gamma_{\mathrm{twist}}=\frac{K_{\mathrm{s}}a}{2\sqrt{3}\pi}\cdot\theta\left\{\frac{2\pi\eta r_0}{\sqrt{3}a}\theta-\ln\left[\sinh\left(\frac{2\pi\eta r_0}{\sqrt{3}a}\theta\right)\right]-\ln 2\right\}+\frac{2\sqrt{3}E_{\mathrm{c}}}{a}\cdot\theta \tag{S29}$$

For a specific twisted bilayer system, K_{s}, a and η are known material constants, while r_0 and E_{c} are assumed to be constant (as established in the previous studies [3, 4]) and can be determined by fitting Eq. (S29) to our atomistic simulation results.

As the twist angle increases, the relative size of AA-node gradually expands. Beyond the critical twist angle ($\theta>\theta_{\mathrm{c}}$), these nodes evolve into AA-domains, accompanied by the formation of a hexagonal network of domain walls (Fig. 1g and h). Figure 1i shows that both AA-domains and domain walls possess relatively higher energy and significantly contribute to the total energy of the twisted lattice. To capture the high-energy features of AA-domains and domain walls, we defined γ_{AA} as the energy density associated with AA-domains and E_{dw} as the energy (per unit length) of domain walls. It is noted that, since the atomic arrangement within AA-domains is not ideally perfect AA stacking, the parameter γ_{AA} should be interpreted conceptually as an average stacking fault energy density. Based on the above analyses, we obtained the following expression for γ_{twist} at large twist angles,

$$\gamma_{\mathrm{twist}}=\frac{E_{\mathrm{dw}}\cdot l_{\mathrm{dw}}+\gamma_{\mathrm{AA}}\cdot S_{\mathrm{AA}}}{S} \tag{S30}$$

where l_{dw} is the total length of domain walls within one superlattice and is equal to 3λ, and S and S_{AA} represent the areas per superlattice and per AA stacking domain, respectively, with $S=2D^2/\sqrt{3}$ and $S_{\mathrm{AA}}=S/3$. We further obtained the following expression for γ_{twist},

$$\gamma_{\mathrm{twist}}=\frac{4\sqrt{3}E_{\mathrm{dw}}}{a}\cdot\sin\left(\frac{\theta}{2}\right)+\frac{1}{3}\gamma_{\mathrm{AA}} \tag{S31}$$

Combining Eq. (S29) and Eq. (S31), we wrote the total energy density of twisted bilayer system with hexagonal symmetry as,

$$\gamma_{\text{twist}} = \begin{cases} \dfrac{K_s a}{2\sqrt{3}\pi} \cdot \theta \left\{ \dfrac{2\pi\eta r_0}{\sqrt{3}a}\theta - \ln\left[\sinh\left(\dfrac{2\pi\eta r_0}{\sqrt{3}a}\theta \right) \right] - \ln 2 \right\} + \dfrac{2\sqrt{3}E_c}{a} \cdot \theta, \ \theta \in [0, \ \theta_c) \\ \dfrac{4\sqrt{3}E_{\text{dw}}}{a} \cdot \sin\left(\dfrac{\theta}{2} \right) + \dfrac{1}{3}\gamma_{\text{AA}}, \ \theta \in [\theta_c, \ \dfrac{\pi}{6}] \end{cases} \tag{S32}$$

where K_s, a and η are known material constants, and r_0, E_c, E_{dw} and γ_{AA} can be obtained by fitting Eq (S32) to atomistic simulation results.

1.3 Derivation of analytical expressions for the local rotation, strain, stress fields, and energy density in twisted trilayer systems.

For the twisted trilayer systems, if the middle layer is rotated by a certain angle with respect to top and bottom layers, then two triangular dislocation networks generate at two twist interfaces between the middle layer and the top/bottom layers, as shown in Figs. 1a and S2 a-c. The middle layer coincides with the (x, z) plane in the coordinate. These is no dislocation network between top and bottom layers due to their complete commensurability. Thus, the entire twisted trilayer system is governed by two dislocation networks that exist between the middle layer and the top/bottom layers. Each dislocation network can be regarded as a twisted bilayer system.

Assuming that the top and bottom layers are only influenced by the nearest dislocation network formed with the middle layer, their local rotation field $\phi_{\text{R}}^{\text{tT-Top/Bot}}$, strain field $\boldsymbol{\varepsilon}^{\text{tT-Top/Bot}}$, and stress field $\boldsymbol{\sigma}^{\text{tT-Top/Bot}}$ are identical to those in twisted bilayer systems and can be written in the following expressions via the coordinate transformation,

$$\begin{aligned} &\phi_{\text{R}}^{\text{tT-Top}}(x,z) = \phi_{\text{T}}^{\text{tB}}\left(x, \frac{h}{2}, z \right) - \frac{\theta}{2} \\ &\boldsymbol{\varepsilon}^{\text{tT-Top}}(x,z) = \boldsymbol{\varepsilon}^{\text{tB}}\left(x, \frac{h}{2}, z \right) \\ &\boldsymbol{\sigma}^{\text{tT-Top}}(x,z) = \boldsymbol{\sigma}^{\text{tB}}\left(x, \frac{h}{2}, z \right) \\ &\phi_{\text{R}}^{\text{tT-Bot}}(x,z) = \phi_{\text{T}}^{\text{tB}}\left(x, \frac{h}{2}, z \right) - \frac{\theta}{2} \\ &\boldsymbol{\varepsilon}^{\text{tT-Bot}}(x,z) = \boldsymbol{\varepsilon}^{\text{tB}}\left(x, \frac{h}{2}, z \right) \\ &\boldsymbol{\sigma}^{\text{tT-Bot}}(x,z) = \boldsymbol{\sigma}^{\text{tB}}\left(x, \frac{h}{2}, z \right) \end{aligned} \tag{S33}$$

where h is the interlayer distance, and $\phi_{\text{T}}^{\text{tB}}$, $\boldsymbol{\varepsilon}^{\text{tB}}$ and $\boldsymbol{\sigma}^{\text{tB}}$ represent the total rotation, strain, and stress fields of top/bottom layer in twisted bilayer systems. By substituting Eqs. (S13), (S15), and (S19) into Eq. (S33), we can obtain the corresponding expressions for rotation, strain and stress fields of top/bottom layers.

The middle layer is influenced by both dislocation networks, and its deformation fields can be obtained through the linear superposition of the two dislocation networks. We determined the local rotation field $\phi_{\mathrm{R}}^{\mathrm{tT\text{-}Mid}}$, strain field $\boldsymbol{\varepsilon}^{\mathrm{tT\text{-}Mid}}$, and stress field $\boldsymbol{\sigma}^{\mathrm{tT\text{-}Mid}}$ in the middle layer as follows,

$$
\begin{aligned}
\phi_{\mathrm{R}}^{\mathrm{tT\text{-}Mid}}(x,z) &= 2\phi_{\mathrm{T}}^{\mathrm{tB}}\left(x,-\frac{h}{2},z\right)+\theta \\
\boldsymbol{\varepsilon}^{\mathrm{tT\text{-}Mid}}(x,z) &= 2\boldsymbol{\varepsilon}^{\mathrm{tB}}\left(x,-\frac{h}{2},z\right) \\
\boldsymbol{\sigma}^{\mathrm{tT\text{-}Mid}}(x,z) &= 2\boldsymbol{\sigma}^{\mathrm{tB}}\left(x,-\frac{h}{2},z\right)
\end{aligned}
\tag{S34}
$$

By substituting Eqs. (S13), (S15), and (S19) into Eq. (S34), we can obtain the corresponding expressions for rotation, strain and stress fields of middle layer.

Similar to twisted bilayer systems, as the twist angle increases, the Moiré-pattern in twisted trilayer systems has a transition from triangular partial-dislocation networks (at small twist angles with $\theta<\theta_c$) to hexagonal domains separating by domain walls (at large twist angles with $\theta>\theta_c$), as shown in Fig. S2. Note that there are two dislocation networks in twisted trilayer system, both total energy and interfacial area of twisted trilayer system are double those of twisted bilayer system. Consequently, the expression for energy density in twist trilayer systems is identical to that in twisted bilayer systems.

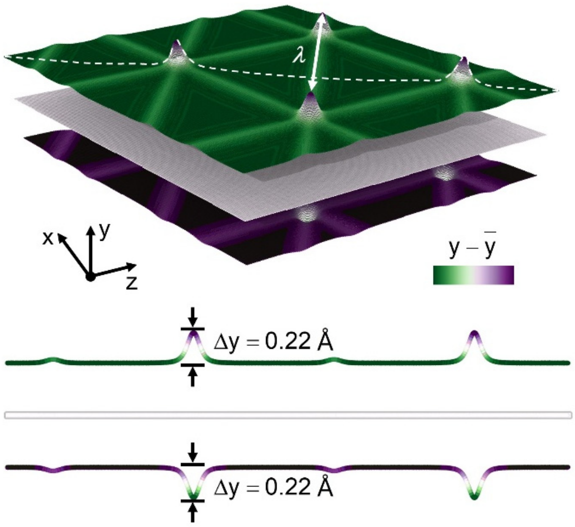

Fig. S1 Out-of-plane displacement of flat mode for tT-G with $\theta = 0.4^\circ$ from atomistic simulations, with a magnification factor of 300.

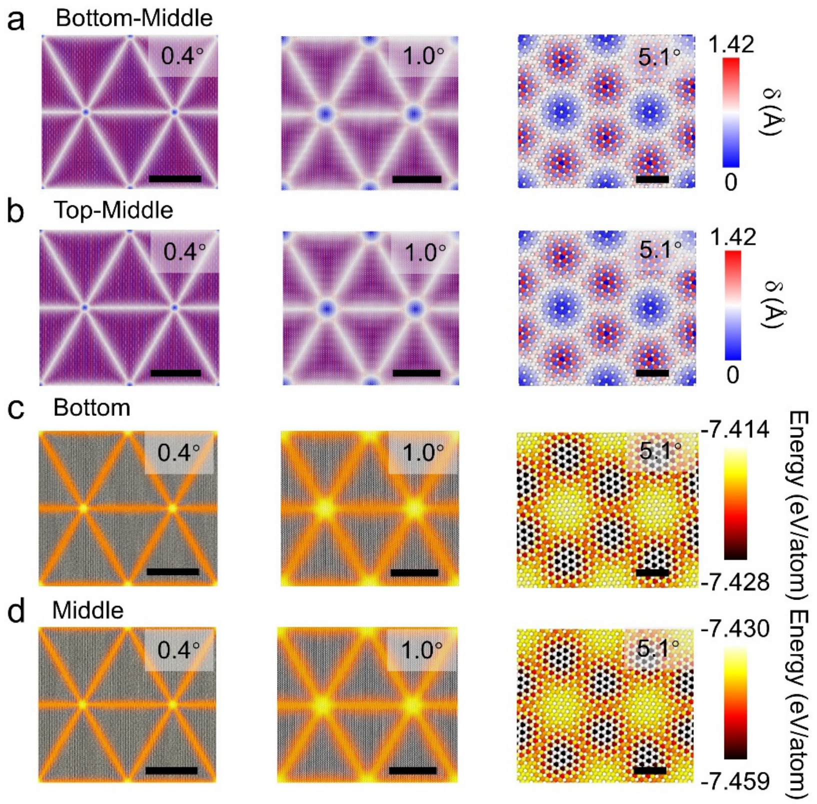

Fig. S2 Lattice mismatch and energy maps in tT-G. **(a-b)** Lattice mismatch in tT-G with varying twist angles, quantified by using δ. The scale bars are 20, 15, and 1 nm from maps with θ= 0.4° to 5.1°, respectively. **(c-d)** Energy maps of tT-G with varying twist angles. The scale bars are 20, 15, and 1 nm from maps with θ= 0.4° to 5.1°, respectively.

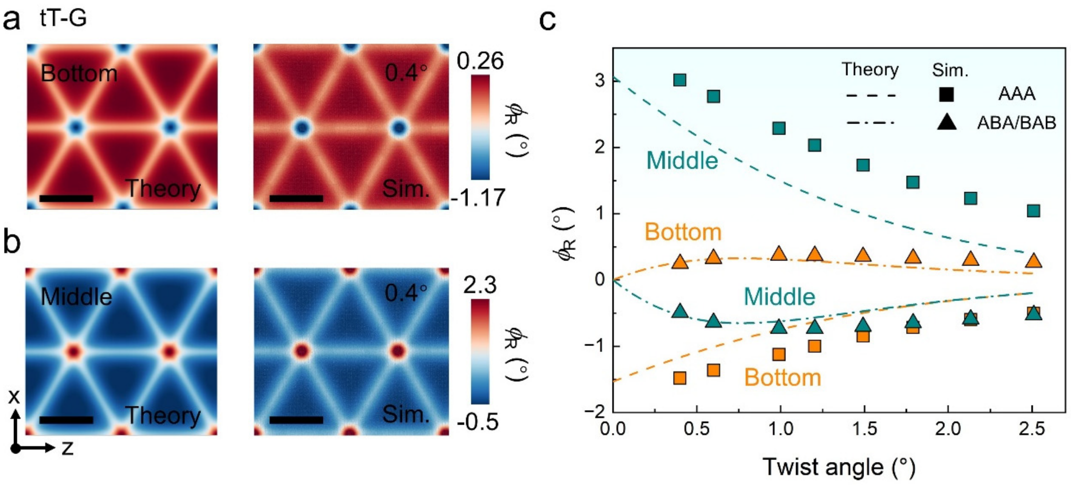

Fig. S3 Local rotation fields in tT-G. **(a-b)** Contours of ϕ_R in the bottom and middle layers of tT-G with θ = 0.4° from theoretical model and atomistic simulations (Sim.). All scale bars are 20 nm. **(c)** ϕ_R at the centers of AAA and ABA/BAB stacking regions as functions of twist angle from theoretical model and atomistic simulations. Data in the bottom and middle layers are in orange and green, respectively.

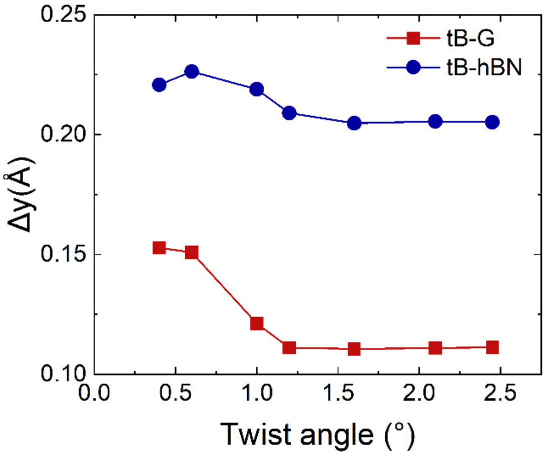

Fig. S4 Out-of-plane displacements Δy in the top layers of tB-G and tB-hBN as functions of the twist angle θ from atomistic simulations.

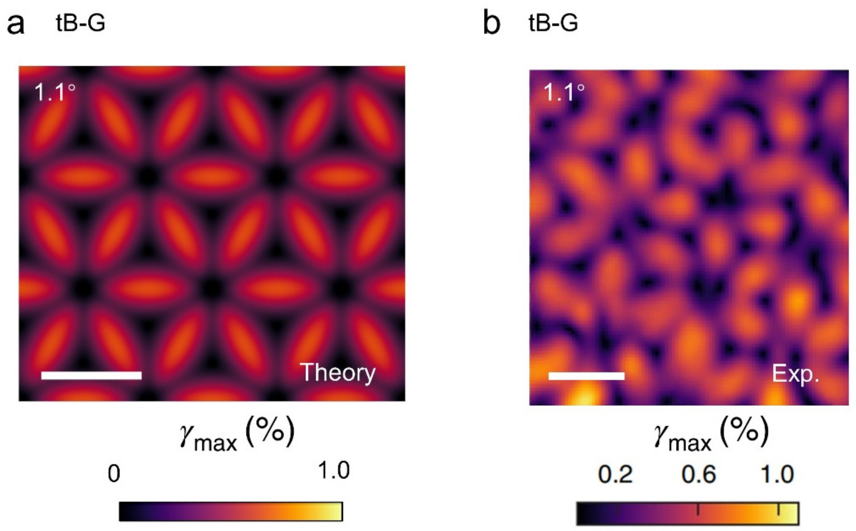

Fig. S5 Maximum in-plane shear strain fields in tB-G. **(a-b)** Maximum in-plane shear strain contours in the top layer of tB-G with θ =1.1° from theoretical model and experiment (Exp.) (Experimental result is reproduced with permission. [5]). Both scale bars are 10 nm.

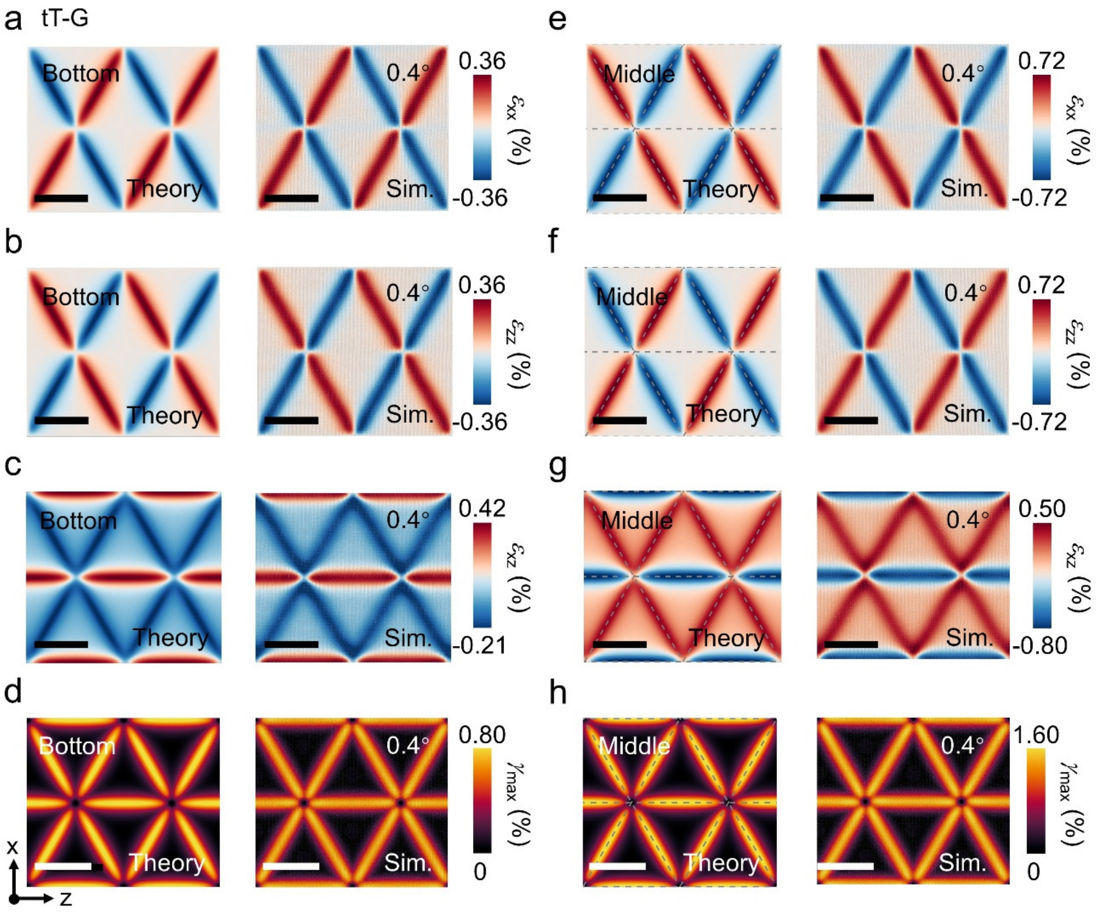

Fig. S6 Strain fields in tT-G. **(a-c)** Contours of in-plane strain components (ε_{xx}, ε_{zz}, and ε_{xz}) in the bottom layer of tT-G with $\theta = 0.4°$ from theoretical model and atomistic simulations. All scale bars are 20 nm. **(d)** Maximum in-plane shear strain contours in the bottom layer of tT-G with $\theta = 0.4°$ from theoretical model and atomistic simulations. Both scale bars are 20 nm. **(e-g)** Contours of in-plane strain components (ε_{xx}, ε_{zz}, and ε_{xz}) in the middle layer of tT-G with $\theta = 0.4°$ from theoretical model and atomistic simulations. All scale bars are 20 nm. **(h)** Maximum in-plane shear strain contours in the middle layer of tT-G with $\theta = 0.4°$ from theoretical model and atomistic simulations. Both scale bars are 20 nm.

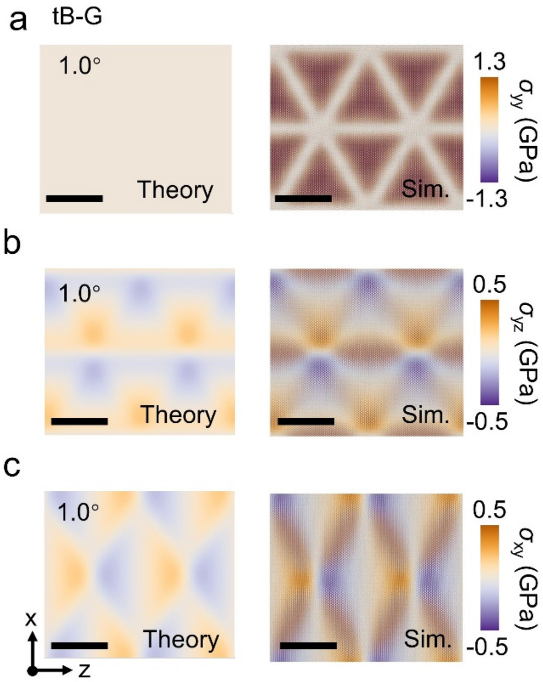

Fig. S7 Out-of-plane stress fields in tB-G. **(a-c)** Contours of out-of-plane stress components (σ_{yy}, σ_{yz}, and σ_{xy}) in the top layer of tB-G with θ=1.0° from theoretical model and atomistic simulations. All scale bars are 15 nm.

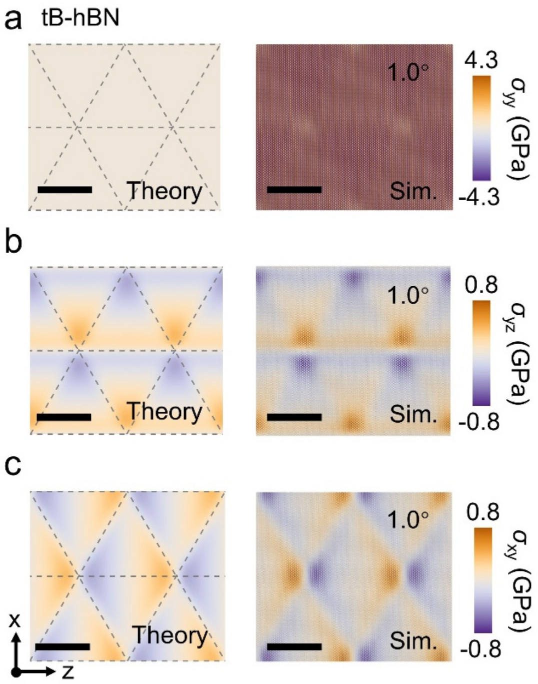

Fig. S8 Out-of-plane stress fields in tB-hBN. **(a-c)** Contours of out-of-plane stress components (σ_{yy}, σ_{yz}, and σ_{xy}) in the top layer of tB-hBN with θ=1.0° from theoretical model and atomistic simulations. All scale bars are 15 nm.

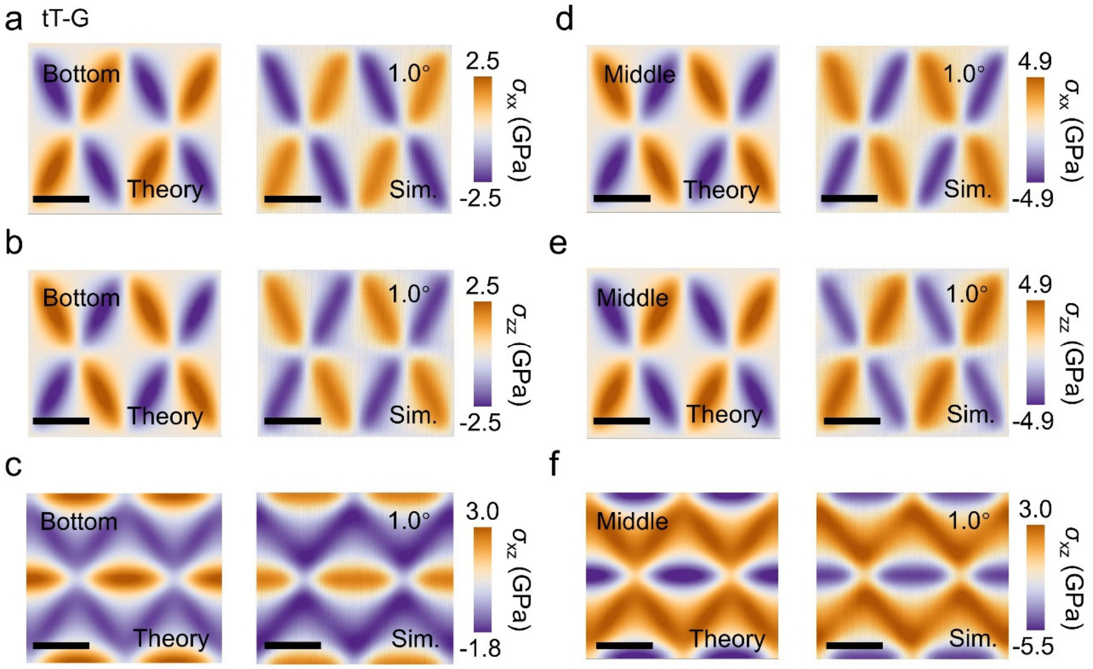

Fig. S9 Stress fields in tT-G. **(a-c)** Contours of in-plane stress components (σ_{xx}, σ_{zz}, and σ_{xz}) in the bottom layer of tT-G with θ= 1.0° from theoretical model and atomistic simulations. All scale bars are 15 nm. **(d-f)** Contours of in-plane stress fields (σ_{xx}, σ_{zz}, and σ_{xz}) in the middle layer of tT-G with θ= 1.0° from theoretical model and atomistic simulations. All scale bars are 15 nm.

Table S1 Elastic constants for Bilayer Graphene and hBN obtained from atomistic simulations

Elastic constants	Bilayer Graphene	Bilayer hBN
C_{11} (GPa)	1057.0	991.6
C_{12} (GPa)	147.8	133.6
C_{13} (GPa)	0.7	1.9
C_{33} (GPa)	16.3	17.7
C_{44} (GPa)	2.0	3.5

2. Atomistic simulations

2.1 Construction of atomic models of tB-G, tB-hBN, and tT-G

To investigate the structural and energetic features of twisted 2D materials, we constructed atomic models of tB-G, tB-hBN, and tT-G according to the geometrical relationships (Fig. S10) of the top and bottom supercells in the twisted 2D bilayer [6]. As shown in Fig. S10, single-layer graphene and hBN with a honeycomb lattice have two primitive vectors, $\boldsymbol{a}_1 = a\left(\sqrt{3}/2, -1/2\right)$ and $\boldsymbol{a}_2 = a\left(\sqrt{3}/2, 1/2\right)$. For a ($q$, p) hexagonal supercell, its basis vectors are expressed as $\boldsymbol{A}_i^{(q,p)} = \sum_j M_{ij}^{(q,p)} \boldsymbol{a}_j \ (i = 1,2)$, where the matrix $M^{(q,p)}$ is given by [6]:

$$M^{(q,p)} = \begin{bmatrix} q & p \\ -p & p+q \end{bmatrix} \tag{S35}$$

Notably, p and q are integers that must obey some specific constraints [6]: they must be distinct and nonzero, possess no common divisor, and the case p-q multiple of 3 must be excluded. Similarly, two basis vectors of the (p, q) supercell are expressed as $\boldsymbol{B}_i^{(p,q)} = \sum_j M_{ij}^{(p,q)} \boldsymbol{a}_j \ (i = 1,2)$. The twist angle between the (q, p) and (p, q) supercells is given by [6]:

$$\tan\theta = \frac{\sqrt{3}\left(p^2 - q^2\right)}{p^2 + q^2 + 4pq} \tag{S36}$$

Thus, the twisted bilayer system containing the (q, p) and (p, q) supercells, along with a twist angle θ, can preserve the long-range translational symmetry.

Following the geometrical relationships of the supercells, we constructed atomic models of tB-G, tB-hBN, and tT-G with specific twist angles θ varying from 0° to 60°. These twist angles and the corresponding values of p and q are listed in Table S2. In this section, we briefly introduce the construction process of twisted 2D materials. We first generated AB-stacked bilayer graphene and hBN with sufficiently large in-plane dimensions, with interlayer spacings of 3.36 Å and 3.42 Å and lattice constant a of 2.46 Å and 2.51 Å, respectively. We then removed the bottom-layer atoms that lie outside the rhombus delineated by the supercell vectors $\boldsymbol{A}_1$ and $\boldsymbol{A}_2$, as well as the top-layer atoms that are outside the rhombus delineated by the supercell vectors $\boldsymbol{B}_1$ and $\boldsymbol{B}_2$. Finally, we obtain the twisted bilayer supercell by rotating the top layer by a given θ with respect to the bottom layer. To ensure statistical significance, we replicated the corresponding supercells at least twice. As a result, the final atomic models of tB-G and tB-hBN contain 13,008 to 163,352 atoms. For periodic tT-G, we replicated tB-G twice along the z-direction to obtain twisted quadrilayer graphene (tQ-G) and then removed the top layer of tQ-G.

2.2 Atomistic simulation details

To investigate the structural and energetic features of twisted 2D materials and further validate our theoretical model, we performed a series of large-scale atomistic simulations for tB-G, tB-hBN, and tT-G with different

twist angles θ. All the simulations were performed via the large-scale atomic/molecular massively parallel simulator (LAMMPS) [7]. We constructed atomic models of tB-G, tB-hBN, and tT-G with the twist angle θ varying from 0° to 60°. During the simulations, we used the second-generation reactive empirical bond order (REBO) potential [8] and Kolmogorov–Crespi (KC) potential [9, 10] to describe the bonded intralayer and nonbonded interlayer interactions of C atoms in tB-G and tT-G, respectively. For the tB-hBN system, we employed the Tersoff-type empirical interatomic potential [11, 12] and interlayer potential (ILP) [10] to describe the bonded intralayer and nonbonded interlayer interactions of B and N atoms, respectively. The REBO potential can accurately describe the energetic features and mechanical properties of various carbon structures [8]. The Tersoff-type potential can accurately describe the experimental structure and phonon dispersion of monolayer hBN [11, 12]. Both the KC potential and the ILP are registry dependent, incorporating long-range vdW attraction and short-range anisotropic repulsion, and can accurately capture the thermodynamic, structural, and mechanical properties of multilayer graphitic and hBN systems, respectively [9, 10]. Throughout the simulations, we imposed periodic boundary conditions along two in-plane directions. All the simulated samples were equilibrated by initial energy minimization with the conjugate gradient (CG) algorithm. Subsequently, the damped dynamics method FIRE [13] and the CG algorithm were used alternatively to relax the simulated systems, with a force convergence criterion of 10^{-6} eV/Å. To ensure complete relaxation and stress release, the "box/relax" keyword was used during the CG minimization. We performed these cycles ten times to ensure that all the simulated samples fully relaxed. The atomic stress tensor of each atom was calculated via the Virial stress theorem [14]. The atomic strain tensor E of each atom was computed based on the current and initial configurations of the simulated sample and was expressed as [15]:

$$\mathbf{E} = \frac{1}{2}\left(\mathbf{F}\mathbf{F}^{\mathrm{T}} - \mathbf{I}\right) \tag{S37}$$

where **I** is the identity matrix, and **F** represents the atomic-level deformation gradient and is expressed by [15]:

$$\mathbf{F} = \left(\sum_{l \in N_k} \mathbf{d}_{lk}^{0\,\mathrm{T}} \mathbf{d}_{lk}\right)^{-1} \left(\sum_{j \in N_k} \mathbf{d}_{lk}^{0\,\mathrm{T}} \mathbf{d}_{lk}\right) \tag{S38}$$

where N_k is the total number of neighboring atoms of atom k and where $\mathbf{d}_{lk}^{0}$ and $\mathbf{d}_{lk}$ are the displacement vectors of atom k relative to its neighboring atom l before and after deformation, respectively. During simulations, the potential energy of each atom is divided into two parts: intralayer energy and interlayer energy. The intralayer potential energy of each atom in graphene and hBN was calculated by using the REBO and Tersoff potentials, respectively. The interlayer potential energy of each atom in graphene and hBN was calculated by using the KC and ILP potentials, respectively. For the few-layer system, the neighboring layers underwent structural relaxation after twisting, leading to changes in the interlayer and intralayer energies compared with those of the initial system with perfect AB stacking. The change in the interlayer energy is referred to as the misfit energy, whereas the change in the intralayer energy is termed the elastic energy. The

elastic and misfit energies of the entire twisted system with a given twist angle were calculated by aggregating the interlayer and intralayer potential energies of all the atoms after simulation and subtracting the corresponding energies of all the atoms of the initial AB-stacked system (without twisting), respectively. The total energy of the simulated system is the sum of the elastic and misfit energies. The average total, elastic and misfit energies per atom were calculated by averaging these energies over all the atoms. The total energy density of the simulated system was calculated by dividing the total energy by the interface area of the twisted system at equilibrium.

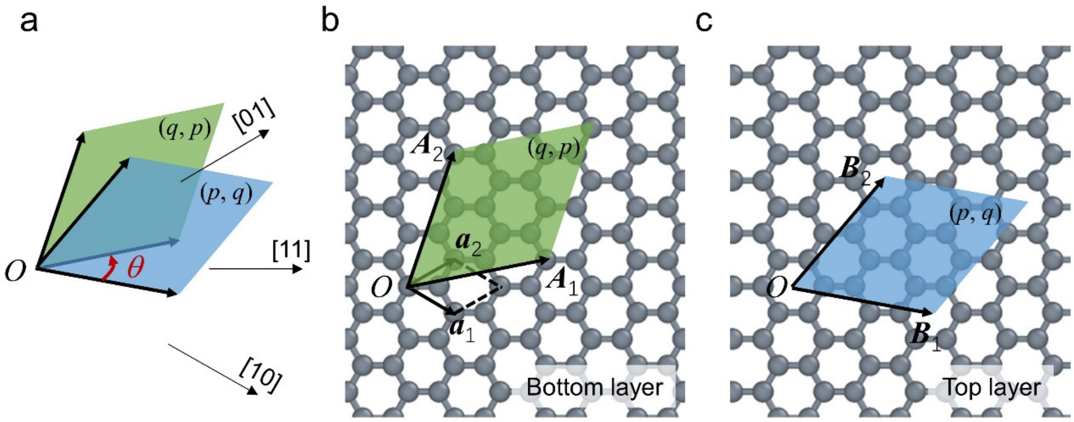

Fig. S10 Schematic illustration of supercells of bottom and top layers of twisted 2D bilayer. **(a)** Graphical representation illustrating the angle θ between bottom (represented by (q, p)) and top (represented by (p, q)) supercells. **(b)** Atomic structure of the supercell in the bottom layer. a_1 and a_2 are two primitive vectors of 2D hexagonal materials. A_1 and A_2 are two basis vectors of the supercell in the bottom layer. **(c)** Atomic structure of the supercell in the top layer. B_1 and B_2 are two basis vectors of the supercell in the top layer. In (B) and (C), the supercells with p=2 and q=1 are two typical examples.

Table S2 Twist angle θ and corresponding values of p and q

θ (°)	(q, p)
0.4	(82, 83)
0.6	(55, 56)
1.0	(32, 33)
1.2	(27, 28)
1.6	(20, 21)
2.0	(16, 17)
2.5	(13, 14)
3.1	(10, 11)
3.9	(8, 9)
4.4	(7, 8)
5.1	(6, 7)
6.0	(5, 6)
7.3	(4, 5)
11.0	(5, 7)
13.2	(2, 3)
15.2	(7, -6)
16.4	(3, 5)
17.9	(6, -5)
21.8	(1, 2)
23.7	(7, 15)
26.0	(3, 7)
27.8	(4, -3)
32.2	(1, 3)
34.0	(10, -7)
36.3	(22, -15)
38.2	(3, -2)
42.1	(1, 5)
43.6	(8, -5)
44.8	(2, 12)
46.8	(10, -6)
49.0	(12, -7)
50.6	(14, -8)
52.7	(18, -10)
54.0	(22, -12)
54.9	(26, -14)
55.6	(15, -8)
56.1	(17, -9)
57.6	(27, -14)
58.0	(33, -17)
58.4	(41, -21)
58.8	(55, -28)

SI References

1. Morse PM, Feshbach H. *Methods of Theoretical Physics*. New York: McGraw-Hill, 1953.

2. Vitek V. On the difference between the misorientation dependences of the energies of tilt and twist boundaries. *Scr Metall* 1987; **21**: 711-714.

3. Read WT, Shockley W. Dislocation models of crystal grain boundaries. *Phys Rev* 1950; **78**: 275-289.

4. Dai S, Xiang Y, Srolovitz DJ. Structure and energy of (1 1 1) low-angle twist boundaries in Al, Cu and Ni. *Acta Mater* 2013; **61**:1327-1337.

5. Kazmierczak NP, Van Winkle M, Ophus C *et al.* Strain fields in twisted bilayer graphene. *Nat Mater* 2021; **20**: 956-963.

6. Latil S, Amara H, Sponza L. Structural classification of boron nitride twisted bilayers and ab initio investigation of their stacking-dependent electronic structure. *SciPost Phys* 2023; **14**: 53.

7. Plimpton S. Fast parallel algorithms for short-range molecular dynamics. *J Comput Phys* 1995; **117**: 1-19.

8. Brenner DW, Shenderova OA, Harrison JA *et al.* A second-generation reactive empirical bond order (REBO) potential energy expression for hydrocarbons. *J Phys: Condens Matter* 2002; **14**: 783-802.

9. Kolmogorov AN, Crespi VH. Registry-dependent interlayer potential for graphitic systems. *Phys Rev B* 2005; **71**: 235415.

10. Ouyang W, Mandelli D, Urbakh M *et al.* Nanoserpents: Graphene nanoribbon motion on two-dimensional hexagonal materials. *Nano Lett* 2018; **18**: 6009-6016.

11. Sevik C, Kinaci A, Haskins JB *et al.* Characterization of thermal transport in low-dimensional boron nitride nanostructures. *Phys Rev B* 2011; **84**: 085409.

12. Kınacı A, Haskins JB, Sevik C *et al.* Thermal conductivity of BN-C nanostructures. *Phys Rev B* 2012; **86**: 115410.

13. Guénolé J, Nöhring WG, Vaid A *et al.* Assessment and optimization of the fast inertial relaxation engine (fire) for energy minimization in atomistic simulations and its implementation in lammps. *Comput Mater Sci* 2020; **175**: 109584.

14. Thompson AP, Plimpton SJ, Mattson W. General formulation of pressure and stress tensor for arbitrary many-body interaction potentials under periodic boundary conditions. *J Chem Phys* 2009; **131**: 154107.

15. Shimizu F, Ogata S, Li J. Theory of shear banding in metallic glasses and molecular dynamics calculations. *Mater Trans* 2007; **48**: 2923-2927.
